# Significant Association of Urinary Toxic Metals and Autism-Related Symptoms—A Nonlinear Statistical Analysis with Cross Validation

**DOI:** 10.1371/journal.pone.0169526

**Published:** 2017-01-09

**Authors:** James Adams, Daniel P. Howsmon, Uwe Kruger, Elizabeth Geis, Eva Gehn, Valeria Fimbres, Elena Pollard, Jessica Mitchell, Julie Ingram, Robert Hellmers, David Quig, Juergen Hahn

**Affiliations:** 1 Arizona State University, Tempe, AZ, United States of America; 2 Rensselaer Polytechnic Institute, Troy, NY, United States of America; 3 Southwest College of Naturopathic Medicine, Tempe, AZ, United States of America; 4 Arizona Allergy Associates, Phoenix, AZ, United States of America; 5 Doctor’s Data, St. Charles, IL, United States of America; Universidad de Jaen, SPAIN

## Abstract

**Introduction:**

A number of previous studies examined a possible association of toxic metals and autism, and over half of those studies suggest that toxic metal levels are different in individuals with Autism Spectrum Disorders (ASD). Additionally, several studies found that those levels correlate with the severity of ASD.

**Methods:**

In order to further investigate these points, this paper performs the most detailed statistical analysis to date of a data set in this field. First morning urine samples were collected from 67 children and adults with ASD and 50 neurotypical controls of similar age and gender. The samples were analyzed to determine the levels of 10 urinary toxic metals (UTM). Autism-related symptoms were assessed with eleven behavioral measures. Statistical analysis was used to distinguish participants on the ASD spectrum and neurotypical participants based upon the UTM data alone. The analysis also included examining the association of autism severity with toxic metal excretion data using linear and nonlinear analysis. “Leave-one-out” cross-validation was used to ensure statistical independence of results.

**Results and Discussion:**

Average excretion levels of several toxic metals (lead, tin, thallium, antimony) were significantly higher in the ASD group. However, ASD classification using univariate statistics proved difficult due to large variability, but nonlinear multivariate statistical analysis significantly improved ASD classification with Type I/II errors of 15% and 18%, respectively. These results clearly indicate that the urinary toxic metal excretion profiles of participants in the ASD group were significantly different from those of the neurotypical participants. Similarly, nonlinear methods determined a significantly stronger association between the behavioral measures and toxic metal excretion. The association was strongest for the Aberrant Behavior Checklist (including subscales on Irritability, Stereotypy, Hyperactivity, and Inappropriate Speech), but significant associations were found for UTM with all eleven autism-related assessments with cross-validation *R*^2^ values ranging from 0.12–0.48.

## Introduction

Many studies have investigated the relationship of toxic metals and toxic chemicals to autism. A very comprehensive review of these studies has recently been published [1]. The majority of studies show a link between environmental toxicants and autism, however, some studies also indicate no association. We found that the analyses conducted in the reviewed papers focused for the most part on linear univariate statistical techniques and, furthermore, an independent validation of the models obtained with different samples was not considered. The challenge with this is that some relationships cannot be appropriately identified using linear univariate techniques and the lack of an independent or cross-validatory model evaluation can result in identification of relationships which are unique to the analyzed data but will not hold up in a broader context. We will address these points in this paper by focusing on nonlinear statistical techniques and, furthermore, validating all results using a cross-validatory model evaluation. The focus of this work is on toxic metals, but it is important to note that there is ample evidence of a similar strong association of ASD with toxic chemicals [1] and the analysis approach used in this paper can also be applied to other data sets.

We first provide a brief overview of the literature on autism toxic metals to put the results from this study into the proper context. One group of studies investigated potential correlations between environmental exposure to toxic metals and prevalence of ASD. For example, one case-control study in San Francisco found that increasing levels of mercury, cadmium, and nickel in the air were associated with a higher prevalence of ASD [2]. Similarly, seven of eight ecological studies found that increasing levels of environmental mercury correlated with an increased prevalence of ASD [3–9], whilst the eighth study found a similar correlation with lead [10].

In addition to the environmental studies, there have been at least 40 case-control studies investigating the level of toxic metals in a total of 2089 individuals with ASD versus 1821 typically-developing controls, including measurements in urine, blood, hair, nails, teeth, and brain samples [1]. Nineteen of these studies (47%) reported elevated levels of one or more toxic metals in children with ASD versus typically-developing controls. Seventeen of the studies (43%) reported similar levels of toxic metals in children with ASD versus controls, but some of these studies included a small number of controls (7 studies had less than 20 controls), so they were probably underpowered, and most of the studies investigated only one or a few toxic metals. Furthermore, none of these studies performed nonlinear multivariable statistical analysis which might be able to find correlations that linear univariate analysis methods may miss.

Some examples of these types of studies that investigated excretion of toxic metals and autism are discussed in the following. Two studies found low levels of mercury in baby hair of children with ASD vs. controls [11, 12], and another study found low levels of several toxic metals in hair of young children with ASD versus controls [13], while another study found low levels of mercury in hair of young children but higher levels in older children [14]; these four studies suggest that studies of hair of infants/young children with ASD need careful interpretation, especially since one of the studies found that level of mercury in baby hair correlated inversely with autism severity; it has been speculated that low levels of mercury in baby hair of children with ASD are related to a detoxification impairment [15].

Several studies have suggested that urinary porphyrin concentrations may be biomarkers of exposure to certain toxic metals [16–18]. This hypothesis is strengthened by two studies that found that treatment with dimercaptosuccinic acid (DMSA), a medication which can enable the body to increase excretion of certain toxic metals, resulted in reductions of porphyrin levels in children with ASD [19, 20]. Seven case-control studies have reported abnormal urinary porphyrin levels in children with ASD versus controls [19–25]. One study found that urinary porphyrins were strong predictors of ASD diagnosis [22], and five studies reported that higher urinary porphyrins were correlated with more severe ASD [19, 21, 23, 26, 27].

Ten studies have reported improvements in ASD symptoms after detoxification treatment, suggesting that the toxic metals may be contributing to the severity of ASD symptoms [28–37]. One of the studies reported a strong correlation between improvement in symptoms and level of toxic metals excreted in the urine during treatment [38]. However, these studies were not placebo-controlled, so a placebo-controlled study is needed to further investigate these observations.

Similarly, eight studies have reported a relationship between autism/IQ and levels of toxic metals, including measurements in urine [15, 38, 39], blood [40], and hair [11, 41–43]. Seven of the eight studies found that increasing levels of toxic metals were correlated with increased ASD symptoms or decreased IQ [15, 38–43]. One study of baby hair reported that low levels of mercury were associated with more severe autism [11], but a replication study did not observe that relationship [12].

There are several mechanisms which can explain at least some of the above mentioned observations: increased intake, increased absorption, or decreased excretion (resulting in an increased body burden). For example, many studies report that children with autism and their mothers have low glutathione [44–47], which is a primary molecule needed for removal of some toxic metals by binding to them and being excreted in the feces. Consequently, low glutathione can result in an increased body burden of toxic metals. Another factor that also decreases the ability to excrete toxic metals in feces is increased use of oral antibiotics [12, 48–51] since oral antibiotics have been shown (in rats) to almost completely inhibit excretion of mercury [52, 53] due to their effect on altering gut flora.

Summarizing, a number of studies suggest that children with autism have several indicators that point towards a higher body burden of a variety of toxic metals. However, one of the limitations of many of the previous studies is that they only investigated one or a few select toxic metals, whereas more recent literature suggests that a number of environmental toxicants may be important [15, 38]. Additionally, the discussed studies focused on relatively simple linear statistical analysis which may be unable to identify existing nonlinear and/or multivariable relationships. Another important issue is a lack of an independent model validation, i.e. a cross-validatory analysis, may result in identification of relationships which may be a result of overfitting and may, therefore, not be representative. These shortcomings can result in an underrepresentation of correlations between toxic metals and ASD (due to the use of simple statistical techniques) as well as an overrepresentation of correlation (due to lack of cross-validation). The presented study seeks to address these limitations by: (a) investigating an extensive set of ten toxic metal and let the analysis determine which, if any, of these may be important/unimportant, (b) including a significant number of participants in the study (67 participants on the spectrum, and 50 controls), (c) evaluating the participants on the spectrum using 11 different autism measures to ensure that results will not just hold up for one measure, (d) using nonlinear extensions of Fisher Discriminant Analysis and Partial Least Squares regression for analysis of the data, and (e) using leave-one-out cross-validation throughout the analysis to ensure statistical independence of the finding which is essential for making reliable predictions.

The purpose of this paper is to conduct an extensive investigation of the toxicological status of children with autism compared to age- and gender-matched neurotypical children. Specifically, we will show that:

1. Individuals with autism have abnormal urinary toxic metal excretion (i.e., higher levels in several cases) as compared to the neurotypical individuals participating in this study, and
2. The severity of autism can be predicted with reasonable accuracy from the urinary toxic metal excretion data.

The analysis is based upon nonlinear statistical analysis techniques which we believe are important because biological systems frequently exhibit nonlinear responses to stimuli; for example, having twice as high a concentration of one toxic metal in the urine does not necessarily result in twice as high a value for an autism measure. Furthermore, cross-validation is used to ensure statistical independence, i.e., that the results do not just hold up for fitting a particular data set but instead that the techniques are able to predict data not used for the analysis.

## Materials and Methods

### Participant selection

The participants reported in this study were part of a 12-month nutrition/dietary treatment study known as the ASU Nutrition/Diet Treatment Study. The data reported in this paper is at baseline, prior to the initiation of treatment. Participants and/or their parents/guardians provided written informed consent and written assent was performed when applicable. This study was approved by the Institutional Review Board of Arizona State University.

The study was advertised by email to approximately 2500 ASD families in Arizona, using the contact list of the Autism Society of Greater Phoenix and the Autism/Asperger’s Research Program at Arizona State University (ASU). Interested ASD families attended a one hour informational meeting, and consenting families joined the study between November 2011 and April 2012. Neurotypical families were recruited from friends of the ASD families and professionals who work with ASD families.

**Enrollment Criteria—ASD Group**
1. Diagnosis of autism spectrum disorder (autism, PDD-NOS, or Asperger’s) by a psychiatrist, psychologist, or developmental pediatrician
2. Verification of diagnosis by ASU staff based on the Autism Diagnostic Observation Schedule (ADOS) and/or Childhood Autism Rating Scale (CARS-2).
3. Age of 2.5 years to 60 years
4. No major changes in behavioral or medical treatments in the previous two months, and no intention to make such changes during the 12 months of the study.
5. No usage of nutritional supplements (vitamins, minerals essential fatty acids, carnitine) or special diets in the previous two months.

**Enrollment Criteria—Neurotypical Group**
1. No diagnosed mental disorders, including autism spectrum disorders, ADHD, depression, anxiety, etc.
2. No first-degree relatives of individuals with ASD (no siblings or parents)
3. Age of 2.5 years to 60 years
4. No usage of nutritional supplements (vitamins, minerals, essential fatty acids, carnitine) or special diets in the previous two months.

#### Participants

The characteristics of the study participants are listed in Table 1. The ASD group and the neurotypical controls have similar age distributions (mostly children, some teens, and a few adults), and similar gender distributions (mostly male). For the ASD group, 100% met the criteria for ASD per the CARS-2, and 88% met the criteria for ASD per the ADOS (Most of the 8 participants who met only the CARS-2 criteria were high-functioning teens/adults. The clinical judgement of the diagnostician was that they were clearly on the ASD spectrum, so they were admitted to the study).

**Table 1:** Characteristics of study participants.

	ASD	Neurotypical
**Total Participants**	67	50
**Male**	55 (82%)	41 (82%)
**Female**	12 (18%)	9 (18%)
**Age (years)**	11.5 ± 8.5	12.2 ± 7.5
Children (ages 3–12)	Children *n* = 48 (72%)	Children *n* = 34 (68%)
Teens (ages 13–20)	Teens *n* = 13 (19%)	Teens *n* = 11 (22%)
Adults (ages 20+)	Adults *n* = 6 (9%)	Adults *n* = 5 (10%)
	Autism = 50 (75%)	
**Diagnosis**	Asperger’s = 8 (12%)	
	PDD-NOS = 7 (10%)	
	Regressive = 22 (33%)	
**Autism Onset**	Plateau = 18 (27%)	
	Early Onset = 25 (37%)	
**Asthma**	17 (25%)	8 (16%)
**Food Allergies**	16 (24%)	2 (4%)
**Other Allergies**	32 (48%)	15 (30%)
**Other Health Issues** (note: these are likely under-reported since we only asked a general question about “other health conditions”, and some of these symptoms might be viewed as part of autism)	ADHD: 10; sensory problems: 4; cerebral palsy: 3; intellectual disability: 2; learning disability: 2; depression: 2; hypotonia: 2; seizures: 2; early puberty: 2; dysphagia; reflux; vascular malformation; spinal fusion; agenesis of lung; gastritis; eczema; apraxia; type 2 diabetes; type 1 diabetes; OCD; anxiety; mood disorder; sleep disorder	nocturnal enuresis; Hashimoto’s thyroiditis

### Biomarker Measurements

First-morning urine samples were collected, immediately frozen at −20°C for up to three days, and shipped frozen overnight on dry ice in a blinded fashion to Doctor’s Data for testing. Doctor’s Data is a commercial laboratory approved by CLIA, the Clinical Laboratory Improvement Amendments program operated by the US Department of Health and Human Services which oversees approximately 200,000 laboratories in the US. Toxic metals were measured using Inductively-Coupled Plasma Mass Spectrometry, and creatinine was measured using the Jaffe method. Urine samples were normalized by creatinine to account for different dilution. For some of the toxic metals, the levels were below the detection limit, so caution needs to be used in interpreting those results—see Table 2 in the Results section. If a measured value was below the detection limit, it was replaced with 2/3 of the detection limit for statistical analysis.

**Table 2:** Level of toxic metals in first-morning urine. Note that for some metals (aluminum, mercury, antimony) results were often below the detection limit, so results for those metals must be interpreted cautiously.

Metal	% below detection limit (Autism/controls)	ASD Average (25th %/75th %) mcg/g-creatinine	Neurotypical Average (25th %/75th %) mcg/g-creatinine	% difference	p-value (from t-test)
Aluminum	31%/46%	9.03 (3.3/11.6)	8.55 (3.3/8.7)	6%	n.s.
Arsenic	0%/0%	13.6 (4.9/13.7)	11.2 (6.3/12.8)	21%	n.s.
Cadmium	0%/0%	0.34 (0.21/0.44)	0.39 (0.21/0.45)	−13%	n.s.
Cesium	0%/0%	4.0 (2.4/5.2)	3.7 (2.6/4.8)	8%	n.s.
Mercury	42%/46%	0.63 (0.20/0.74)	0.53 (0.20/0.61)	19%	n.s.
Nickel	0%/0%	4.3 (2.5/5.9)	4.0 (2.5/4.6)	8%	n.s.
Lead	3%/6%	0.59 (0.26/0.76)	0.35 (0.22/0.47)	72%	0.0001
Antimony	45%/54%	0.088 (0.04/0.10)	0.059 (0.04/0.07)	49%	0.02
Tin	10%/14%	2.7 (0.38/2.9)	0.99 (0.3/1.3)	177%	0.003
Thallium	0%/0%	0.17 (0.10/0.20)	0.11 (0.08/0.13)	50%	0.0001
Tungsten	1%/0%	0.29 (0.14/0.35)	0.25 (0.14/0.32)	0%	n.s.

### Autism Severity and Overall Functioning Assessments

A large number of assessments of autism severity and overall functioning were conducted, some by a professional evaluator and some by the parents. The professional evaluations included

- Autism Diagnostic Observation Schedule (ADOS, [54]): We calculated the sum of the communication and social scores, using both the full 0–3 scale (Raw ADOS) and the adjusted 0–2 scale (Adj ADOS) in which scores of “3” are counted as “2” for diagnostic purposes.
- Childhood Autism Rating Scale (CARS-2, [55]): We calculated the total, using either the Standard or High-Functioning form, as appropriate.
- Severity of Autism Scale (SAS, [38]): The SAS is a single number on a scale of 0–10 to evaluate overall severity of autism symptoms. It was evaluated after the ADOS and CARS-2 by the professional evaluator (PRO-SAS). It was also evaluated independently by the parent (SAS-Parent).

All of the ADOS, CARS-2, and SAS evaluations were done by the same evaluator (either EP or JI).

Parents (or the participants in a few cases for high-functioning adults) completed an initial medical history form, several questionnaires to assess autism and related symptoms, including the following:

- Aberrant Behavior Checklist (ABC, [56]): We calculated the total of the five subscales.
- Autism Treatment Evaluation Checklist (ATEC, [57]): We calculated the total of the four subscales.
- Pervasive Developmental Disorders Behavior Inventory (PDD-BI): We calculated a modified Autism Composite [29].
- Severity of Autism Scale (SAS-Parent), as discussed above
- Social Responsiveness Scale (SRS, [58]): We calculated the total of all the subscales.
- Short Sensory Profile (SSP, [59]): We calculated the total of all the subscales.
- PGI-R2 is an expanded version of the PGI-R [47]. The PGI-R2-Initial evaluates initial symptom severity in 17 areas (using a scale of none = 0, mild = 1, moderate = 2, severe = 3), and an Average is calculated based on the score of all 17 areas.

### Statistical Analysis

All statistical analysis was performed by author-developed MATLAB code. A summary of these techniques is presented here with more detailed information provided in S1–S5 Appendices. Additionally, all raw data used in this study is provided in S1–S4 Tables.

#### Multivariate non-causal modeling techniques (classification and analysis)

The aim of this part of the study is to evaluate whether it is possible to diagnose autism based on the excretion of toxic metals in urine using Fisher discrimination analysis. This involves linear Fisher discriminant analysis (FDA) and its nonlinear counterpart termed kernel FDA (KFDA). FDA is a multivariate projection based technique that aims to determine the best separation between two or more clusters of samples [60]. More precisely, FDA determines a projection direction such that the orthogonal projections of the samples of different clusters are best separated. In other words, the centers of each cluster are projected onto this line to have the optimal distance from each other. For this study, we have a total of 67 participants that are diagnosed to be on the autism spectrum and 50 participants that are neurotypical. The sample of each participant includes measurements of 10 urine toxins. This requires normalizing the combined set of 117 samples, i.e. center the samples for each of the 10 variables to have a mean of zero and a variance of one. This is followed by computing the mean vector for the 67 samples of participants on the spectrum and the 50 samples of neurotypical participants. The projection of the difference in mean of both groups describes the between cluster, or group, variation. The second aspect is to consider the within cluster variation, described by the covariance matrices of both clusters. Nonlinear extensions to FDA have been proposed if the different classes cannot be separated effectively by a linear projection of the samples of both classes [61, 62]. S1 and S2 Appendices present more detailed descriptions of FDA and its nonlinear counterpart KFDA, respectively.

To examine commonality among the various autism measures, we also consider the application of principal component analysis (PCA). In a similar fashion to FDA, PCA determines projections of the 67 samples of participants on the autism spectrum onto directions such that these projections describe a maximum variance for each direction [63–66]. The technique, consequently, extracts variation from the multivariate data set that describes a maximum amount of information in each direction. If the variables within the multivariate set possess a significant degree of correlation, the first few such principal components capture most of the information, whilst the remaining lower order components are uninformative. More precisely, the first few dominant components capture the underlying variable interrelationships (correlation), which reveal variable clusters that show a similar correlation structure. In other words, PCA can reveal subsets of variables that describe common features within the multivariate set of autism measures. S3 Appendix presents a more detailed treatment concerning the working of PCA.

#### Multivariate causal modeling techniques (regression)

The aim of this part of the study is to determine if severity of autism and related symptoms can be predicted based on excretion of toxic metals in urine, using regression. Partial least squares is selected for this task, as it is a linear regression technique tailored to applications involving relatively small numbers of samples. Such a scenario is common in many application areas, including chemometrics and medicine, where in addition to a small sample size, the number of random variables can be significant. Given that the data sets involves data from 67 participants on the spectrum, each containing the measured concentration of 10 different urine toxins and various autism measures we have such a scenario, necessitating the use of PLS. Höskuldsson [67] pointed out that PLS provides more stable predictors in such scenarios, compared to other multivariate regression techniques, such as ordinary least squares, maximum redundancy, or canonical correlation regression. More precisely, the strength of PLS is that it does not require a matrix inversion to determine a linear regression model. This follows from the property of PLS to maximize a covariance criterion between a linear combination of a set of cause, or predictor, and the linear combination of a set of effect, or response, variables [66, 68, 69].

Defining the set of predictor and response variables as *x* and *y*, respectively, a linear regression model is given by *y* = *Bx* + *e*, where *e* are model residuals and *B* is an unknown regression matrix. Here, the random vectors *x* and *y* contain the urine toxins and one or more of the autism measures, respectively. Instead of using a standard regression to determine *B*, PLS defines one projection, or direction, vector for the 67 samples of the urine toxins *x* and one direction vector for the 67 samples of the autism measures *y*. The projections of the samples of *x* and *y* onto their respective directions are then used to determine the regression model. This guarantees that important information that is encapsulated within the random vectors *x* and *y* is utilized in constructing a regression model. After determining the first set of these directions, the impact of the projections is then subtracted from *x* and *y*, allowing the determination of further directions. Compared to ordinary least squares, the PLS regression has advantages when significant noise and error ratios are present or high correlation exists amongst the variable set *x*. This is achieved by omitting less important and uninformative projection directions and only including those that produce a significant contribution to the prediction of *y*. For each variable combination evaluated in this work, models with one to the number of original variables were evaluated and the final number of latent variables was chosen to maximize the cross-validated *R*^2^. S4 Appendix contains a more detailed treatment of the PLS algorithm. The basic linear PLS technique has also been augmented to model nonlinear relationships between *x* and *y*, i.e. *y* = *f*(*x*) + *e*, where *f*(…) is a smooth nonlinear function. S5 Appendix discusses kernel PLS, a popular nonlinear extension of PLS.

#### Cross Validation (model validation)

It is essential to validate the performance of a regression model to ensure that it does not only perform well on the data set used for model identification, but instead can reliably be used to predict outcomes that were not used for model fitting. For large sample to variable ratios, this can be accomplished by removing a portion of the samples, identify a regression model on this reduced set and validate its performance on the omitted samples. This guarantees a statistically independent validation of the model performance [70]. If the sample size is small, however, model validation presents a problem, as omitting a portion of the data may yield a significant reduction in the sample numbers [71]. In addition, removing specific samples may have an undesired effect upon model identification and validation. With a total of 67 independent samples, each containing 10 urine toxins, we have a small sample size. To adequately validate the model performance in such scenarios, a cross validation approach can be considered [72], such as leave-one-out cross validation.

Leave-one-out cross-validation removes the first sample from the data set, identifies a model utilizing the remaining 66 samples and examines the performance of the identified model on this first sample. The performance, i.e., the modeling error for this sample, is then stored. This is followed by removing the second sample, identifying a new model from the remaining 66 samples and again, computing the modeling error for the second sample. In fact, each sample is removed once and, in turn, a total of 67 models are identified that are respectively applied to the sample left out for each case. As the validation is statistically independent from the model identification, cross validation is a statistically sound method to evaluate model performance [73]. It should finally be noted that cross validation assists in determining the optimal model complexity, i.e. how many different and, more importantly, which urine toxins affect various autism measures. Upon determining the optimal model complexity, the final step is to identify a model based on the optimal model structure using all samples.

The criteria for assessing the performance of a regression model, the *R*^2^ statistic is often considered, which is defined as *R*^2^ = 1 − *SS*_*e*_/*SS*_*y*_. Here, *SS* represents the sum of squares for the model residuals, *e*, and the samples of the response variable, *y*. More precisely, $SS_{e}=\sum_{i=1}^{n}e_{-i}^{2}$ and $SS_{y}=\sum_{k=1}^{n}y_{k}^{2}$, where *e*_−*i*_ is the residual of the *i*th sample that is not included in the set used to identify the model (leave-one-out cross-validation). It should be noted that the largest value that this statistic can assume is 1 (perfect model) and values that are close to zero or negative indicate a model that poorly predicts the response variable.

#### Kernel density estimation (descriptive statistics)

This technique is used to distinguish participants in the ASD group from those of the neurotypical group. It estimates the probability density function of a random variable using a set of reference samples. The core idea is that additional samples are located most likely close to the reference samples [74–76]. In order to formulate this idea into an algorithm, each reference sample is associated with a density function that centers on the sample. The sum of these density functions, or kernel functions, then represents the estimated probability density function. Potential kernel functions are Gaussian, triangular, Epanechnikov or uniform functions and contain a parameter to adjust their shape and are of the form $\frac{1}{h}K\left(\frac{x-x_{i}}{h}\right)$, where *x* is an additional sample, *x*_*i*_ is the *i*th reference sample and *h* is the adjustment parameter. The estimated density function is then $\hat{f}\left(x\right)=\frac{1}{nh}\sum_{i=1}^{n}K\left(\frac{x-x_{i}}{h}\right)$, where *n* is the number of reference samples. The parameter *h* can be obtained by minimizing the mismatch between the unknown density function of the random variable *x*, *f*(*x*), and the estimated density function $\hat{f}\left(x\right)$ using the mean integrated squared error $MISE\left(h\right)=\int_{-\infty }^{\infty }\left(f\left(x\right)-\hat{f}\left(x\right)\right)dx$. The MISE objective function can be evaluated using a cross validatory criterion [74].

## Results

### Levels of Urinary Toxic Metals

**Fig 1:**
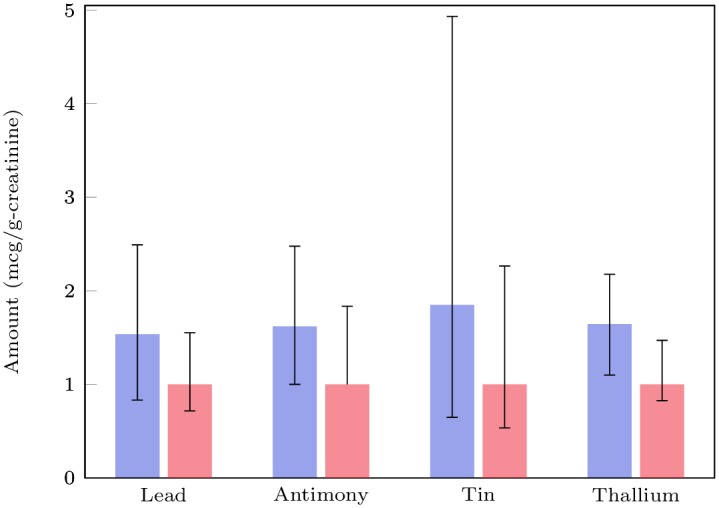
Median values of urinary toxic metals for ASD and control groups, normalized to the median of the control values. The bars represent the 25th and 75th percentiles.

The heavy metals excretion data were compared between the neurotypical participants and the participants on the autism spectrum. When using single variable statistics, there are several metals for which a statistically significant difference between the two groups can be observed (see Table 2). For example, the average excretion rates of lead, tin, thallium, and antimony are 72%, 174%, 50%, and 49% greater for the ASD group than the neurotypical group leading, respectively, with p-values of 0.001, 0.007, 0.0003, and 0.02, respectively. However, there is large variability in each group, and overlap between groups, so it is not possible to classify participant as ASD or neurotypical with a significant degree of certainty using univariate statistics only (see Fig 1).

### Diagnosing Autism

Given the limitations of univariate statistical methods, we next used multivariable analysis methods to try to develop a method to diagnose ASD. Multivariable statistics looked at the differences in toxic metal excretion data by simultaneously taking all metals into account. Hotelling’s T^2^ test, the multivariate equivalent of the popular Student’s t-test, was used to evaluate statistical differences between the group on the autism spectrum and that diagnosed as neurotypical. The p-value was 9.56e-4, indicating statistically significant differences between the groups; however, this only examines changes in the average and does not take the large variance of each variable into account. Classification into one of the two groups, i.e., on the spectrum or neurotypical, was still not possible with a significant degree of certainty by directly using the UTM excretion values due to large variability in each group. To overcome this deficiency, a discriminant analysis and estimation of the probability density functions was used to take changes in the mean as well as variation in the data into account

**Fig 2:**
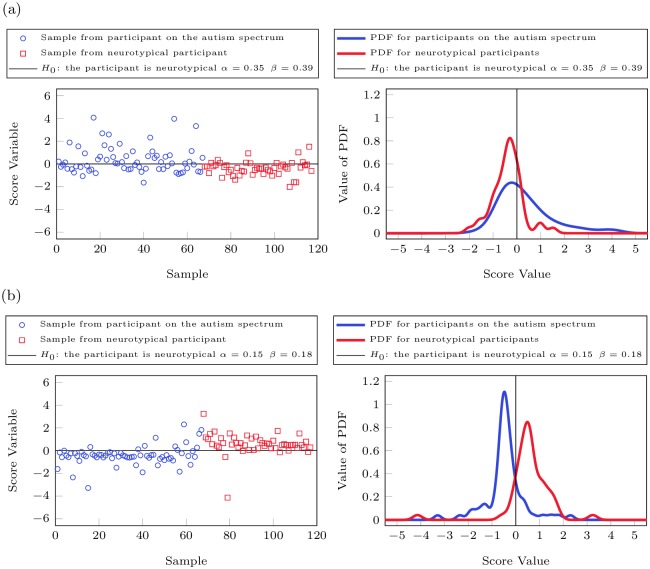
Fisher Discriminant Analysis of urine toxic metal data. Fig 2(a) shows the score variables and the PDF of the neurotypical participants and the participants on the autism spectrum using FDA while Fig 2(b) contains the same information derived by KFDA. The groups of the neurotypical participants and the participants on the spectrum have different distributions, however, there is significant overlap between the two groups when linear FDA is used. While there is still overlap between the two groups even for KFDA, the distributions becomes more distinct when nonlinear statistical techniques such as KFDA are used.

Fisher Discriminant Analysis [60], including cross-validation, was employed to determine differences between the two groups. While there is a clear difference in the distribution of the data from neurotypical participants and those on the spectrum, there is also significant overlap (see Fig 2(a)). As such, it is not possible to achieve a low Type I error (incorrectly diagnosing a neurotypical participant as ASD) and a low Type II error (incorrectly diagnosing a participant with ASD as neurotypical) for this data set using FDA, as the best separation would result in a Type I error of 0.35 and a Type II error of 0.39. However, when Kernel Fisher Discriminant Analysis (KFDA), which is a nonlinear extension of FDA, was employed then a better separation could be achieved as a Type I error of 0.15 and Type II errors of 0.18 were computed for this data set (see Fig 2(b)). It should be noted that analysis with setting the Type I error to 0.1 were also conducted, however, these produced significant Type II errors for the linear (0.57) and the nonlinear case (0.35) and an approach that determines a trade-off between the Type I and Type II errors was employed instead. A summary of Type I and Type II errors for FDA and KFDA is provided in Table 3 below.

**Table 3:** Type I and Type II errors for classification of participant data into neurotypical participants and participants on the autism spectrum. Type II errors increase as smaller values are chosen for Type I errors. KFDA outperforms its linear counterpart, FDA, for all cases. Only cross-validation results are shown.

	Fisher Discriminant Analysis							Kernel Fisher Discriminant Analysis						
Type I error	0.40	0.35	0.30	0.25	0.20	0.15	0.10	0.40	0.35	0.3	0.25	0.20	0.15	0.10
Type II error	0.36	0.39	0.42	0.45	0.48	0.52	0.57	0.05	0.05	0.06	0.08	0.11	0.18	0.35

Even though classification of the data sets into neurotypical participants and participants on the spectrum is challenging, it nevertheless can be clearly seen from the probability density function (PDF) in Fig 2(b) that there is a distinct difference between the two groups based upon their urine metal excretions if nonlinear analysis techniques are used.

### Predicting Severity of Autism and Related Symptoms

Next, we focused on the data generated by the group of participants on the ASD spectrum in order to analyze correlations between the data and the degree of autism severity. Results for the ABC are discussed first since they had the strongest correlation, and correlations with other measures are discussed later in this document.

Both linear regression and nonlinear regression, via PLS and KPLS, respectively, were performed on the data set. We varied the number of metals to be included in the predictor set from 1 to 10 (all metals) and determined the correlation between the metals data and the autism severity as given by the total ABC score. Furthermore, we looked at every single combination of metals possible for the analysis and performed leave-one-out cross-validation on the results to ensure that the results are statistically independent to avoid overfitting. Furthermore, results for linear regression without cross-validation are also provided in Table 4 to highlight that cross-validation is needed to avoid overfitting as otherwise the adjusted *R*^2^ values will continue to increase or at least reach a plateau at a high level as more input variables are used. All other results in this work, aside from the 3rd column in Table 4, are based upon leave-one-out cross-validation

**Table 4:** Prediction of ABC Total. Correlation between ABC Total value and metal excretion using linear regression (no cross-validation & cross-validation) as well as nonlinear regression (cross-validation). Only the results for the highest *R*^2^ values are shown, but other combinations of metals frequently had similar results.

# Variables	Linear Model			Nonlinear Model	
	Metal Combinations	Max *R*^2^ value		Metal Combinations	Max *R*^2^ value
		No Cross-Validation	Cross-Validation		
1	Cs	0.194	0.175	Cs	0.185
2	Cs/Hg	0.227	0.182	Cs/Hg	0.233
3	As/Cs/Sn	0.265	0.188	Cs/Ni/Sn	0.326
4	**As/Cs/Ni/Sn**	0.296	**0.192**	As/Hg/Ni/Sn	0.367
5	As/Cs/Hg/Ni/Sn	0.318	0.186	Cs/Hg/Ni/Pb/Sn	0.416
6	As/Cs/Hg/Ni/Sn /W	0.329	0.187	Cd/Cs/Hg/Ni/Pb/Sn	0.449
7	As/Cs/Hg/Ni/Sn /Tl/W	0.337	0.165	As/Cd/Cs/Hg/Ni/Pb/Sn	0.471
8	As/Cs/Hg/Ni/Pb/Sn /Tl/W	0.345	0.148	**As/Cd/Cs/Hg/Ni/Pb/Sn/Tl**	**0.475**
9	Al/As/Cs/Hg/Ni/Pb/Sn/Tl/W	0.345	0.118	As/Cd/Cs/Hg/Ni/Pb/Sn/Tl/W	0.406
10	all	0.345	0.062	All	0.327

A summary of the best results for each number of metals, the respective metals used, and the *R*^2^ as determined by cross-validation are shown in Table 4 below. It should be noted that the adjusted *R*^2^ values tend to increase to a certain point as more metals are used as inputs for the regression, but then the *R*^2^ decreases from a certain point on as the model is overfitting the data. This type of analysis result is common when cross-validation is used whereas regression techniques that do not make use of cross-validation tend to provide larger *R*^2^ values as more inputs are added to the model. Furthermore, it should be noted that *R*^2^ values derived from cross-validation tend to be significantly lower than *R*^2^ values derived from simply fitting the regression model (see Table 4) and that it is possible that *R*^2^ of cross-validation can be negative if a model cannot predict the data well.

It can be seen that linear regression results in an *R*^2^ that is 0.192 when four metals are used, whereas the *R*^2^ for nonlinear regression can be as high as 0.475 for eight metals. That being said, nonlinear regression, even for just six metals as inputs, can result in *R*^2^ values of 0.449 which shows a significant correlation between metals excretion and ABC score.

Given the significant correlation between metal excretion and autism severity, we decided to perform a regression analysis of metal excretion against the submeasures that make up the ABC. Similarly to what was done for regression analysis of ABC, all combinations of metals for all numbers of investigated metals have been looked at. A summary of the best results, as measured by *R*^2^ for cross-validation, for each case is shown in Table 5 below.

**Table 5:** Prediction of ABC Total. Correlation between ABC Total value and metal excretion using linear regression (no cross-validation & cross-validation) as well as nonlinear regression (cross-validation). Only the results for the highest *R*^2^ values are shown, but other combinations of metals frequently had similar results.

	# Variables	Linear Model		Nonlinear Model	
		Metal Combinations	Max *R*^2^ value	Metal Combinations	Max *R*^2^ value
Irritability	1	Cs	0.257	Cs	0.254
	2	**Cs/Hg**	**0.300**	Cs/Hg	0.306
	3	As/Cs/Hg	0.293	Hg/Ni/Sn	0.378
	4	As/Cs/Hg/W	0.280	As/Hg/Ni/Sn	0.401
	5	As/Cs/Hg/Ni/W	0.264	Cd/Cs/Hg/Ni/Sn	0.428
	6	As/Cs/Hg/Ni/Tl/W	0.244	**As/Cd/Hg/Ni/Sn/W**	**0.490**
	7	Al/As/Cs/Hg/Ni/Tl/W	0.223	As/Cd/Cs/Hg/Ni/Pb/Sn	0.477
	8	Al/As/Cd/Cs/Hg/Ni/Tl/W	0.190	Al/As/Cd/Cs/Hg/Ni/Pb/Sn	0.411
	9	Al/As/Cd/Cs/Hg/Ni/Sn/Tl/W	0.147	Al/As/Cd/Cs/Hg/Ni Pb/Sn/W	0.359
	10	All metals included	0.095	All metals included	0.252
Lethargy	1	As	0.06	As	0.04
	2	**As/Ni**	**0.087**	As/Ni	0.098
	3	As/Ni/Sn	0.082	**Al/As/Ni**	**0.160**
	4	As/Ni/Sn/W	0.072	As/Cd/Ni/Pb	0.142
	5	As/Hg/Ni/Sn/W	0.058	Al/Cd/Cs/Hg/Ni	0.126
	6	As/Hg/Ni/Pb/Tl/W	0.041	Al/As/Cd/Ni/Pb/W	0.105
	7	As/Hg/Ni/Pb/Sn/Tl/W	0.029	Al/As/Cd/Ni/Pb/Tl/W	0.079
	8	Al/As/Hg/Ni/Pb/Sn/Tl/W	0.008	Al/As/Cd/Cs/Ni/Pb/Tl/W	0.038
	9	Al/As/Cs/Hg/Ni/Pb/Sn/Tl/W	−0.028	Al/As/Cd/Hg/Ni/Pb/Sn/Tl/W	0.015
	10	All metals included	−0.096	All metals included	−0.005
Stereotypy	1	Ni	0.067	Cs	0.060
	2	As/Ni	0.081	Cs/Hg	0.138
	3	Ni/Pb/W	0.091	Cd/Cs/Hg	0.173
	4	As/Ni/Pb/W	0.104	As/Hg/Ni/W	0.356
	5	As/Hg/Ni/Sn/W	0.058	Al/As/Hg/Ni/W	0.347
	6	**Cs/Hg/Ni/Pb/Tl/W**	**0.129**	As/Cd/Cs/Hg/Ni/Sn	0.398
	7	As/Cs/Hg/Ni/Pb/Tl/W	0.122	Al/As/Cd/Cs/Hg/Ni/Pb	0.418
	8	Al/As/Cs/Hg/Ni/Pb/Tl/W	0.110	**Al/As/Cd/Cs/Hg/Ni/Pb/Sn**	**0.430**
	9	Al/As/Cs/Hg/Ni/Pb/Sn/Tl/W	0.083	Al/As/Cd/Cs/Hg/Ni/Pb/Sn/W	0.430
	10	All metals included	0.032	All metals included	0.315
Hyperactivity	1	Cs	0.251	Sn	0.370
	2	**Cs/Sn**	**0.266**	Cs/Sn	0.431
	3	Cs/Sn/W	0.256	Cs/Pb/Sn	0.482
	4	Cs/Sn/Tl/W	0.238	Cd/Cs/Pb/Sn	0.520
	5	Cs/Ni/Sn/Tl/W	0.216	Cd/Cs/Pb/Sn/W	0.542
	6	As/Cs/Ni/Sn/Tl/W	0.189	**Cs/Hg/Ni/Pb/Sn/Tl**	**0.587**
	7	Al/As/Cs/Ni/Sn/Tl/W	0.164	Cd/Cs/Hg/Pb/Sn/Tl/W	0.560
	8	Al/As/Cs/Ni/Pb/Sn/Tl/W	0.137	As/Cd/Cs/Hg/Ni/Pb/Sn/Tl	0.522
	9	Al/As/Cs/Hg/Ni/Pb/Sn/Tl/W	0.091	As/Cd/Cs/Hg/Ni/Pb/Sn/Tl/W	0.452
	10	All metals included	0.035	All metals included	0.345
Inappropriate Speech	1	Cs	0.035	Sn	0.102
	2	**Cs/W**	**0.045**	Sn/W	0.145
	3	Cs/Tl/W	0.033	Sn/Tl/W	0.231
	4	Cs/Pb/Tl/W	0.018	Cd/Pb/Sn/W	0.341
	5	As/Cs/Pb/Tl/W	−0.001	**Cs/Hg/Ni/Pb/Sn**	**0.416**
	6	As/Cs/Pb/Sn/Tl/W	−0.028	Al/As/Cs/Ni/Pb/W	0.203
	7	As/Cs/Hg/Ni/Sn/Tl/W	−0.057	As/Cs/Hg/Ni/Sn/Tl/W	0.157
	8	As/Cs/Hg/Ni/Pb/Sn/Tl/W	−0.086	As/Cd/Cs/Hg/Ni/Sn/Tl/W	0.105
	9	As/Cd/Cs/Hg/Ni/Pb/Sn/Tl/W	−0.134	As/Cd/Cs/Hg/Ni/Pb/Sn/Tl/W	0.058
	10	All metals included	−0.185	All metals included	0.020

The general trend in the results is that nonlinear regression outperforms linear regression for all cases. Also, the best results for linear regression can be found for smaller number of metals investigated, i.e., in all but one case the optimum number of metals is two; in comparison to that nonlinear regression tends to make use of the excretion data involving a larger number of metals for optimal prediction accuracy. Most importantly, results for Irritability (*R*^2^ of 0.490), Stereotypy (*R*^2^ of 0.430), Hyperactivity (*R*^2^ of 0.587), and Inappropriate Speech (*R*^2^ of 0.416) show a large degree of correlation with metal excretion. The only ABC submeasure with only a relatively low correlation is Lethargy (*R*^2^ of 0.160).

Even though only the regression results for the ABC were discussed so far, similar regression analyses (both linear and nonlinear, including cross-validation for both to determine *R*^2^) have been performed for a number of other autism-related measures. The results show correlations with metal excretion, albeit the correlations are not as strong as the results for ABC reported here. Specifically, the best regression results for the effect of metal excretion on autism severity were in the 0.2–0.4 range for *R*^2^ for the ATEC, PDD-BI, PGI-R2, SRS, and CARS-2 measures, while the best results for *R*^2^ for Adj ADOS, Pro SAS, Raw ADOS, SSP, and SAS were in the 0.1–0.2 range. In comparison, the best results for ABC had an *R*^2^ of 0.475. A summary of the results can be found in Table 6 below.

**Table 6:** Regression results for metal excretion on different ASD measures. Only the best number and combination of metals are shown. *R*^2^ results are computed via cross-validation.

ASD Measure	Best R^2^	Excreted Metal Combination Resulting in Largest R^2^
ABC Total	0.475	As/Cd/Cs/Hg/Ni/Pb/Sn/Tl
ABC Total (best 5 inputs)	0.416	Cs/Hg/Ni/Pb/Sn
PDD-BI	0.321	Cs/Hg/Ni/Sn/W
CARS-2	0.258	Cd/Hg/Sn/Tl/W
PGI-R2	0.237	As/Cs/Hg/Ni/Sn
ATEC Total	0.235	Cs/Hg/Ni/W
SRS	0.225	As/Hg/Ni/Sn/W
SSP	0.157	As/Cs/Hg/Ni/W
SAS-Parent	0.142	As/Cs/Hg/Pb
Pro SAS	0.141	As/Hg/Pb/W
Raw ADOS	0.151	Cd/Cs/Ni/Pb/Sn/Tl
Adj. ADOS	0.118	As/Cd/Cs/Ni

**Fig 3:**
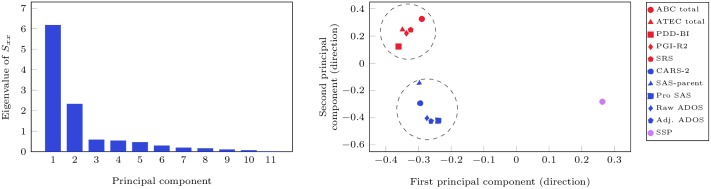
Principal Component Analysis performed for the different autism measures. The Figure on the left shows that two principal components can capture the majority of the differences between the different measures. The analysis shown on the right indicates that there are two clusters with five measures each (ABC, ATEC, PDD-BI, PGI-R2, and SRS; CARS-2, SAS-Parent, Pro SAS, Raw ADOS, Adj ADOS), where there is a high correlation between the autism measures. The measure SSP seems to be distinct from either cluster.

Given that there are significant difference in the *R*^2^ values of the association between metal excretion and autism severity for the different measures, we performed principal component analysis just on the different ASD measures to check if there is consistency between the measures, e.g., if the same participant has a high value using one measure but a low value using another measure, then it may be difficult or even impossible to have both results being correctly reflected in a regression model. The results of this analysis are shown in Fig 3 below.

It can be seen that there are four other measures which are closely correlated with ABC. Specifically, these are ATEC Total, PDD-BI, PGI-R2, and SRS. All of these measures have *R*^2^ values above 0.2, whereas all but one of the other measures which poorly correlated with ABC have *R*^2^ values that are substantially less than 0.2. This analysis can serve as another indicator that the measures which are significantly correlated with the ABC measures also result in good results for regression analysis of metal excretion data against autism severity.

## Discussion

### Levels of Toxic Metals

Higher levels of toxic metals in urine suggest an increased exposure, increased absorption, and/or possibly decreased fecal excretion (most toxic metals are conjugated to glutathione, excreted in the bile into the intestines, and then expelled in the feces). Higher amounts of toxic metals in the urine are suggestive of higher exposure or higher body burden, so the results of this study suggest that a subset of children with autism possibly have higher exposure or higher body burden of lead, tin, thallium, and antimony. These results are similar to results of a previous study by our group which also measured urinary toxic metals using the same methodology in a different set of 55 children with ASD and 44 neurotypical controls, all ages 5–16 years, and also from Arizona. The autism group had higher urinary levels of lead (+74%, p = 0.02), thallium (+77%, p = 0.0001), tin (+155%, p = 0.01), and tungsten (+44%, p = 0.00005). So, the findings of increased lead, thallium, and tin are consistent with the findings in this study, although there is some inconsistency regarding nickel, antimony, and tungsten. Overall, this study represents the first attempt to replicate the findings of increased urinary toxic metals in a different population in the same geographic locale.

A few other studies have also examined urinary excretion of toxic metals in children with ASD versus controls. A study by Blaurock-Busch [77] in Saudi Arabia of 25 children with ASD compared to 25 neurotypical children found significantly increased levels of aluminum, barium, cesium, mercury, and lead. The findings of increased levels of toxic metals is generally consistent with the present study, although there are inconsistencies as to which toxic metals are higher as well as the overall levels of toxic metal excretion, possibly due to differences in age and geographic location.

A study by Wright et al. [78] in the United Kingdom investigated urinary levels of several toxic metals in 56 children with ASD compared to 121 neurotypical children, and did not find significant differences in levels of mercury, antimony, cadmium, or lead. The results for mercury and cadmium are consistent with the present study, but the results for antimony and lead are not.

A study by Albizzatti et al. [79] did not find significant differences in measurements of lead, mercury, cadmium and aluminum in urine from 17 children with ASD compared to 20 neuropsychiatric patients, all in Italy. However, the small sample size suggests that the study was underpowered, and the use of participants who were not typically developing limits the interpretation of the results.

Overall, the finding of increased levels of several toxic metals in urine of children with ASD agrees with two previous studies [15, 77], but disagrees with one large study [78] and one small study [79]. This disagreement is similar to that of the 40 case-control studies discussed in the introduction, in which about half observed levels of increased toxic metals.

However, it should be pointed out that the discussion in this subsection has solely focused on univariate statistical measures. Multivariable statistical techniques and especially techniques that can deal with nonlinear behaviors can reveal additional features contained in a data set as is shown in this paper. While we performed a very detailed analysis using these sophisticated techniques on our data set, we do not have access to the raw data of these other studies and it may be possible that they would show similar results that would be revealed by nonlinear multivariable statistical techniques.

### Autism Diagnosis

The analysis of the data from the neurotypical group and the participants on the spectrum shows that there are clear statistical differences between the two groups. These differences exist even if only a few key measurements are looked at, i.e., single variable statistics such as comparing the excretion average across the group of tin, lead, or thallium, but they become more pronounced when multivariable statistics such as (Kernel) Fisher Discriminant Analysis (Fig 2) are used. While it is possible to achieve a Type 1 error of 0.1 for classification, i.e., 90% of neurotypical children are correctly identified as such, the corresponding Type II error, i.e., the chance that a participant on the spectrum is falsely assumed to be neurotypical, is reasonably large at 0.57. This is especially so when cross-validation is used as Type II errors tend to be higher for cross-validation as the data points used for validation have not been used for determining the analysis. As such, using linear statistical methods on only metal excretion data to determine if a participant is neurotypical or on the spectrum is not recommended due to the large Type II error. When nonlinear techniques such as KFDA were employed then a separation between the two groups of participants becomes more pronounced. Type I errors of 0.1 result in Type II errors of 0.35; however, a better trade-off is to allow Type I errors 0.15 and Type II errors of 0.18, i.e., 85% of the neurotypical patients are correctly identified as such while the chance that a participant on the spectrum is falsely assumed to be neurotypical, is found to be 18% (Fig 2(b)). It is clear from this analysis that there is a statistical difference in the urine metal excretion data between the two groups, but classification on UTM data alone is not recommended as 15% (neurotypical)/18% (ASD) of the patients are incorrectly classified based upon this data set.

### ABC and Toxic Metals

The analysis of the data clearly shows that there is a significant correlation between the ABC, as well as several subcategories of this measure, and metal excretion as measured in this study. The highest correlation is when the excretion data of eight metals (As/Cd/Cs/Hg/Ni/Pb/Sn/Tl) are used to predict the ABC measure. The *R*^2^ value, even though it was determined by cross-validation, which tends to result in lower *R*^2^ values than those for simple regression that are the norm in most papers, was as high as 0.475 in this case. Even for the case of using only five metals (Cs/Hg/Ni/Pb/Sn), the *R*^2^ was still 0.416. It should be noted that the listed combinations of metals are not the only ones that show high correlations; there are several other combinations which resulted in *R*^2^ values that were almost as high as the ones reported here. As such, there does not seem to be any one or two metals that are responsible for the results shown here; that being said, Cs, Hg, and Sn tend to show up in a lot of the combinations of metals that correlated well with ABC values. Furthermore, a sensitivity analysis was conducted for the best set of inputs and the ABC measure. This analysis set the values of all inputs at the average value and then varied one input at a time. The changes of the predicted ABC measure to changes in each input confirm the nonlinear relationship between the inputs and output (results not shown).

Given the high correlation between ABC and metal excretion, the investigation of how metal excretion is correlated with the submeasures that make up ABC also yielded some very strong correlations. Irritability (*R*^2^ of 0.49), stereotypy (*R*^2^ of 0.43), hyperactivity (*R*^2^ of 0.587), and inappropriate speech (*R*^2^ of 0.416) all were strongly correlated with metal excretion. In particular, hyperactivity was strongly correlated as using even only the Sn excretion value showed a correlation of 0.37 with hyperactivity. The only ABC submeasure which did not return strong correlations was lethargy, but even for this measure a weaker correlation of 0.16 was found. Summarizing, the autism measure ABC as a whole, as well as its subcategories showed significant correlations with the metal excretions measured as part of this study.

### Other ASD Measures and Toxic Metals

Investigation with other autism measures also showed correlations with metal excretions, albeit less strong. ATEC, PDD-BI, PGI-R2, SRS, and CARS-2 all had correlations with *R*^2^ of 0.22–0.32 for the best case. In order to corroborate why some measures correlate well while others do not, principal component analysis of the different autism measures have been conducted for the group of all participants on the spectrum. It was found that five of the six measures with *R*^2^ above 0.2 (ABC, ATEC, PD–BI, PGI–R2, and SRS) actually have highly correlated outcomes, whereas the other autism measures do not correlate well with these five, i.e., one measure may be high for one participant while another measure for the same participant may be low. As such, the reduced correlations between metal excretion and the autism measures can be partially explained because the measures are not always consistent. One possible explanation for why ABC correlates particularly strongly with metal excretion, while other measures show less of a correlation, is that metal excretion as used by this study was only measured at one point in time. Similarly, the submeasures that make up ABC (hyperactivity, irritability, etc.) are behaviors that are also assessed at one point in time and this point in time is close to the one where metal excretion was measured. Regardless of what is the cause and the effect here, it can be seen that there is a strong correlation between patterns in the metal excretion and hyperactive behavior occurring at the same point in time. On the other hand, other autism measures include categories which measure skills that are acquired over longer periods of time. For example, the number of words used in speech or social skills are skills that develop over months and years. It is not surprising that measures that include these categories show less of a correlation with a urine toxic metal test that was only done on one particular day. In future studies it would be interesting to investigate metal excretion over an extended period of time and correlate this dynamics of metal excretion to skills that are acquired over the same time period.

### Association or Causation

The strong association between toxic metal excretion and some autism-related scores does not necessarily imply causation. However, toxic metals are by definition known to be detrimental to human health, and a higher body burden of toxic metals would seem likely to worsen autism and related symptoms [15]. The complexity of urine analysis of toxic metals is that whereas high excretion is generally associated with high exposure or uptake, lower excretion may be due to low exposure and/or decreased detoxification ability. Similarly, high values of excretion for one toxic metal may result in a saturation effect such that further increases of excretion of this or other metals have negligible effects on the autism severity; in other words, doubling the excretion may not correlate with a doubling of symptom severity. This complexity may explain why nonlinear regression analysis yielded much more significant results compared to the conceptually simpler linear regression.

It is important to note that while genetics is known to play a major role in autism, that there is also a significant environmental component [80]. The correlations found in this paper can be used as a starting point for further investigations into environmental factors that affect or at least are correlated with autism as there clearly is a correlation between autism and heavy metal excretion (regardless of if this is correlative or causative in nature). Specific areas for further investigation are to determine if there is relationship between autism and heavy metal intake, autism and heavy metal absorption, or autism and reduced detoxification capability. It should be noted that the latter two areas could potentially also be affected by genetics.

### Advantage of Nonlinear Regression

While most of the discussion in this work has focused on the results, one of the key contributions of this work is to use tools from engineering/statistics, such as nonlinear regression and cross-validation, on physiological data. As such, a discussion of the pros and cons of the methods used and the results that they produce is warranted. One of the advantages of using nonlinear regression techniques, such as KPLS, is that complex correlations can be captured in the data. For example, if a participant has extremely low excretion of toxic metals then this can be an indicator of impaired detoxification; the case where this is the result of a very low exposure is ignored for the sake of the argument made in this paragraph. Similarly, if a participant has very high excretion of toxic metals then this can potentially be an indicator of high body burden, resulting from either past exposure/uptake or impaired detoxification in the past. The ideal situation would be where the excretion of toxic metals is always equal to the intake. However, a linear model will not be able to describe this situation as linearity of a model does not allow that lower excretion may be problematic and that higher excretion may also be an indicator of potential problems. Only nonlinear techniques are able to describe data sets where such trends can be found.

One result of this ability to identify more complex behavior in the data set is that nonlinear regression techniques tend to produce higher *R*^2^ values than linear regression, i.e., in the worst case the nonlinear regression should reduce to the linear case. All the results shown in this paper, as well as the thousands of different cases (not shown) where we performed linear/nonlinear regression for all number of metals and metal excretion combinations for the eleven different autism measures investigated in this study reflect this point as linear regression has not outperformed nonlinear regression in a single case. The one note of caution is that nonlinear regression can result in models with more parameters than linear regression and as such, one has to be careful that no overfitting occurs, i.e., where the model reflects the investigated data well but will perform poorly on data other than the one used for the regression. We used cross-validation to ensure that overfitting would not be an issue here by guaranteeing statistical independence for model validation. While it is generally recommended to use cross-validation even for linear models/analysis, the use of cross-validation is especially important for nonlinear models/analysis as nonlinear models tend to include more parameters than linear models.

### Cross-Validation

Leave-one-out (LOO) cross-validation ensures statistical independence of the analysis results as the key is not to find the best regression model for the data, but to find a regression model that can predict data not used for the regression (and then repeat this procedure for all data points by leaving out a different data point at each step). This technique has been extensively used in statistics and engineering [72]. It should be noted that *R*^2^ for cross-validation will almost always be significantly lower than *R*^2^ values for regression as regression only fits a model to the data and does not try to predict, see the comparison between linear regression with and without cross-validation in Table 3 as one example of this; however, prediction (and not fitting!) is the ultimate goal when analyzing data as results should carry over to other data sets and future studies. One point to note is that *R*^2^ values for fitting are almost always considerably larger than *R*^2^ values from cross-validation (which can even be negative for poor predictions). As such, the high *R*^2^ values reported in this work, coupled with the fact that these are *R*^2^ for cross-validation, makes a very strong case that these correlations exist not just for the investigated data set but would likely also be confirmed in other studies.

### Limitations of the Present Study

1. The study was conducted with participants from Arizona, so the differences seen in toxic metal excretion in the ASD group versus the neurotypical group may be specific to the toxic metals present in that environment. Different parts of the United States, or different parts of the world, have different levels of toxic metals in their environment, so the association between toxic metal excretion and ASD severity may vary from region to region. However, the nonlinear analysis approach relating the urinary toxic metal excretion data to ASD severity is likely robust as evidenced by the cross-validation results.
2. We did not assess the underlying reason for the abnormal urinary toxic metal excretion results, e.g., glutathione status and/or environmental exposure of the individuals in the study. This would be important to do in future studies, since abnormal glutathione and/or environmental exposure likely explains some of the abnormal toxic metal excretion in the children with ASD.
3. Urinary levels of toxic metals are a reasonable measure of very recent exposure to toxic metals (last few days), but may not correlate with levels during fetal and early infant development. They may also have only a weak correlation with present levels in the brain, which is probably the most critical area for ASD symptoms. However, they have the advantage of being easily and non-invasively obtained.
4. Some individuals with ASD exhibit pica (eating non-food objects) that may increase their intake of toxic metals. This behavior was not assessed in this study. However, a previous study found that symptoms of pica did not significantly change levels of toxic metals in hair of children with ASD [49].

## Conclusions

This work performed a detailed statistical analysis of urinary toxic metal excretion data and various autism measures. The study involved 117 participants, 67 with an ASD diagnosis and 50 who were neurotypical. One urine sample was collected for each participant and analyzed with respect to 10 toxic metals. Concurrently with the data collected, participants were evaluated using 11 different autism measures.

Our statistical analysis shows that there is a clear difference between the metal excretion data of the participants with and without an autism diagnosis. However, variations within each group were significant which made a clear separation between the groups challenging using single or multivariable linear statistical techniques. KFDA allowed for a reasonably good separation that resulted in Type I errors of 0.15 while Type II errors were also only 0.18. While these errors are too large to be used for diagnostic purposes by themselves, nevertheless they confirm that, at least for the participants of this study, there is a clear difference in the metal excretion data between neurotypical participants and those on the spectrum.

The investigation further focused on determining correlations between toxic metal excretion data and autism severity using linear (PLS) as well as nonlinear techniques (KPLS). A strong correlation was found for the ABC and even more so for some of the submeasures of which the ABC is composed. Reasonably significant correlations between toxic metal excretion and autism severity were also found for all other 10 autism measures investigated here. While no one particular metal stood out as having a much stronger impact on autism severity than others, Cs, Hg, and Sn showed up in a lot of the combinations of metals that correlated well with a number of autism measures and in particular with ABC values.

Lastly, we want to emphasize that one of the contributions of this work was to use state of the art statistical analysis techniques on the data set from this study to determine how regression results hold up when leave-one-out cross-validation is used in addition to investigating resulting from using nonlinear versus linear analysis techniques. While linear regression can be used to arrive at similar conclusions to what is stated here, using nonlinear techniques revealed much stronger correlation and classification results, even when cross-validation is used.

## Supporting Information

S1 Appendix Details of the linear FDA. (PDF)

S2 Appendix Nonlinear kernel FDA (KFDA) algorithm. (PDF)

S3 Appendix Principal Component Analysis (PCA). (PDF)

S4 Appendix Details of the linear PLS algorithm. (PDF)

S5 Appendix Details of the nonlinear KPLS algorithm. (PDF)

S1 Table Raw urine toxic element data for participants on the autism spectrum. (PDF)

S2 Table Raw urine toxic element data for neurotypical participants. (PDF)

S3 Table ABC total and submeasure scores for participants on the autism spectrum. (PDF)

S4 Table Additional measures of autism severity for participants on the autism spectrum. (PDF)

## References

[1] Rossignol DA, Genuis SJ, Frye RE. Environmental toxicants and autism spectrum disorders: a systematic review. Translational Psychiatry. 2014;4(2):1–23. 10.1038/tp.2014.4 24518398 PMC3944636

[2] Windham GC, Zhang L, Gunier R, Croen LA, Grether JK. Autism Spectrum Disorders in Relation to Distribution of Hazardous Air Pollutants in the San Francisco Bay Area. Environmental Health Perspectives. 2006;114(9):1438–1444. 10.1289/ehp.9120 16966102 PMC1570060

[3] Bartell SM, Lewandowski TA. Administrative Censoring in Ecological Analyses of Autism and a Bayesian Solution. Journal of Environmental and Public Health. 2011 5;2011:1–5. 10.1155/2011/202783 21647346 PMC3103873

[4] Blanchard KS, Palmer RF, Stein Z. The value of ecologic studies: mercury concentration in ambient air and the risk of autism. Reviews on Environmental Health. 2011;26(2):111–118. 10.1515/reveh.2011.015 21905454

[5] DeSoto MC, Hitlan RT. Fish Consumption Advisories and the Surprising Relationship to Prevalence Rate of Developmental Disability as Reported by Public Schools. Journal of Environmental Protection. 2012;03(11):1579–1589. 10.4236/jep.2012.311174

[6] Lewandowski TA, Bartell SM, Yager JW, Levin L. An Evaluation of Surrogate Chemical Exposure Measures and Autism Prevalence in Texas. Journal of Toxicology and Environmental Health, Part A. 2009;72(24):1592–1603. 10.1080/15287390903232483 20077234

[7] Palmer RF, Blanchard S, Stein Z, Mandell D, Miller C. Environmental mercury release, special education rates, and autism disorder: an ecological study of Texas. Health & Place. 2006;12(2):203–209. 10.1016/j.healthplace.2004.11.005 16338635

[8] Palmer RF, Blanchard S, Wood R. Proximity to point sources of environmental mercury release as a predictor of autism prevalence. Health & Place. 2009;15(1):18–24. 10.1016/j.healthplace.2008.02.001 18353703

[9] Rury J. Links between Environmental Mercury, Special Education, and Autism in Louisiana. Baton Rouge, Louisiana; 2006.

[10] Schweikert C, Li Y, Dayya D, Yens D, Torrents M, Hsu DF. Analysis of Autism Prevalence and Neurotoxins Using Combinatorial Fusion and Association Rule Mining. In: Ninth IEEE International Conference on Bioinformatics and BioEngineering; 2009. p. 400–404.

[11] Holmes AS, Blaxill MF, Haley BE. Reduced Levels of Mercury in First Baby Haircuts of Autistic Children. International Journal of Toxicology. 2003;22(4):277–285. 10.1080/10915810305120 12933322

[12] Adams JB, Romdalvik J, Levine KE, Hu LW. Mercury in first-cut baby hair of children with autism versus typically-developing children. Toxicological & Environmental Chemistry. 2008;90(4):739–753.

[13] Kern JK, Grannemann BD, Trivedi MH, Adams JB. Sulfhydryl-Reactive Metals in Autism. Journal of Toxicology and Environmental Health, Part A. 2007;70(8):715–721. 10.1080/15287390601188060 17365626

[14] Majewska MD, Urbanowicz E, Rok-Bujko P, Namyslowska I, Mierzejewski P. Age-dependent lower or higher levels of hair mercury in autistic children than in healthy controls. Acta neurobiologiae experimentalis. 2010;70(2):196–208. 20628443 10.55782/ane-2010-1791

[15] Adams JB, Audhya T, McDonough-Means S, Rubin RA, Quig D, Geis E, et al Toxicological Status of Children with Autism vs. Neurotypical Children and the Association with Autism Severity. Biological Trace Element Research. 2012;151(2):171–180. 10.1007/s12011-012-9551-1 23192845

[16] Heyer NJ, Bittner AC Jr, Echeverria D, Woods JS. A cascade analysis of the interaction of mercury and coproporphyrinogen oxidase (CPOX) polymorphism on the heme biosynthetic pathway and porphyrin production. Toxicology Letters. 2006;161(2):159–166. 10.1016/j.toxlet.2005.09.005 16214298

[17] Pingree SD, Simmonds PL, Rummel KT, Woods JS. Quantitative Evaluation of Urinary Porphyrins as a Measure of Kidney Mercury Content and Mercury Body Burden during Prolonged Methylmercury Exposure in Rats. Toxicological Sciences. 2001;61(2):234–240. 10.1093/toxsci/61.2.234 11353132

[18] Woods JS. Porphyrin Metabolism as Indicator of Metal Exposure and Toxicity In: Goyer RA, Cherian MG, editors. Toxicology of Metals. No. 115 in Handbook of Experimental Pharmacology. Springer Berlin Heidelberg; 1995 p. 19–52. 10.1007/978-3-642-79162-8_2

[19] Nataf R, Skorupka C, Amet L, Lam A, Springbett A, Lathe R. Porphyrinuria in childhood autistic disorder: Implications for environmental toxicity. Toxicology and Applied Pharmacology. 2006;214(2):99–108. 10.1016/j.taap.2006.04.008 16782144

[20] Geier DA, Geier MR. A Prospective Study of Mercury Toxicity Biomarkers in Autistic Spectrum Disorders. Journal of Toxicology and Environmental Health, Part A. 2007;70(20):1723–1730. 10.1080/15287390701457712 17885929

[21] Geier DA, Geier MR. A prospective assessment of porphyrins in autistic disorders: A potential marker for heavy metal exposure. Neurotoxicity Research. 2006;10(1):57–63. 10.1007/BF03033334 17000470

[22] Heyer NJ, Echeverria D, Woods JS. Disordered Porphyrin Metabolism: A Potential Biological Marker for Autism Risk Assessment. Autism Research. 2012;5(2):84–92. 10.1002/aur.236 22298513 PMC3329579

[23] Kern JK, Geier DA, Adams JB, Mehta JA, Grannemann BD, Geier MR. Toxicity biomarkers in autism spectrum disorder: A blinded study of urinary porphyrins. Pediatrics International. 2011;53(2):147–153. 10.1111/j.1442-200X.2010.03196.x 20626635

[24] Woods JS, Armel SE, Fulton DI, Allen J, Wessels K, Simmonds PL, et al Urinary Porphyrin Excretion in Neurotypical and Autistic Children. Environmental Health Perspectives. 2010;118(10):1450–1457. 10.1289/ehp.0901713 20576582 PMC2957928

[25] Youn SI, Jin SH, Kim SH, Lim S. Porphyrinuria in Korean Children with Autism: Correlation with Oxidative Stress. Journal of Toxicology and Environmental Health, Part A. 2010;73(10):701–710. 10.1080/15287391003614000 20391113

[26] Geier DA, Kern JK, Garver CR, Adams JB, Audhya T, Nataf R, et al Biomarkers of environmental toxicity and susceptibility in autism. Journal of the Neurological Sciences. 2009;280:101–108. 10.1016/j.jns.2008.08.021 18817931

[27] Geier DA, Kern JK, Geier MR. A Prospective Blinded Evaluation of Urinary Porphyrins Verses the Clinical Severity of Autism Spectrum Disorders. Journal of Toxicology and Environmental Health, Part A. 2009;72(24):1585–1591. 10.1080/15287390903232475 20077233

[28] Accardo P, Whitman B, Caul J, Rolfe U. Autism and Plumbism: A Possible Association. Clinical Pediatrics. 1988;1(27):41–44. 10.1177/000992288802700108 2446813

[29] Adams JB, Baral M, Geis E, Mitchell J, Ingram J, Hensley A, et al Safety and efficacy of oral DMSA therapy for children with autism spectrum disorders: Part B—Behavioral results. BMC Clinical Pharmacology. 2009;9(17). 10.1186/1472-6904-9-17 19852790 PMC2770991

[30] Blaurock-Busch E, Amin OR, Dessoki HH, Rabah T. Efficacy of DMSA Therapy in a Sample of Arab Children with Autistic Spectrum Disorder. Maedica. 2012;7(3):214–221. 23400264 PMC3566884

[31] Cohen DJ, Johnson WT, Caparulo BK. Pica and elevated blood lead level in autistic and atypical children. American Journal of Diseases of Children. 1976;130(1):47–48. 10.1001/archpedi.1976.02120020049007 813517

[32] Geier DA, Geier MR. A clinical trial of combined anti-androgen and anti-heavy metal therapy in autistic disorders. Neuro Endocrinology Letters. 2006;27(6):833–838. 17187010

[33] Eppright TD, Sanfacon JA, Horwitz EA. Attention deficit hyperactivity disorder, infantile autism, and elevated blood-lead: a possible relationship. Missouri medicine. 1996;93(3):136–138. 8867271

[34] Goin-Kochel RP, Mackintosh VH, Myers BJ. Parental reports on the efficacy of treatments and therapies for their children with autism spectrum disorders. Research in Autism Spectrum Disorders. 2009;3(2):528–537. 10.1016/j.rasd.2008.11.001

[35] Kidd PM. Autism, an extreme challenge to integrative medicine. Part II: Medical management. Alternative Medicine Review. 2002;7(6):472–499. 12495373

[36] Lonsdale D, Shamberger RJ, Audhya T. Treatment of autism spectrum children with thiamine tetrahydrofurfuryl disulfide: A pilot study. Neuroendocrinology Letters. 2002;23:303–308. 12195231

[37] Patel K, Curtis LT. A Comprehensive Approach to Treating Autism and Attention-Deficit Hyperactivity Disorder: A Prepilot Study. The Journal of Alternative and Complementary Medicine. 2007;13(10):1091–1098. 10.1089/acm.2007.0611 18166120

[38] Adams JB, Baral M, Geis E, Mitchell J, Ingram J, Hensley A, et al The Severity of Autism Is Associated with Toxic Metal Body Burden and Red Blood Cell Glutathione Levels. Journal of Toxicology, Journal of Toxicology. 2009;p. 1–7. 10.1155/2009/532640 20107587 PMC2809421

[39] Blaurock-Busch E, Amin OR, Dessoki HH, Rabah T. Toxic Metals and Essential Elements in Hair and Severity of Symptoms among Children with Autism. Maedica. 2012;7(1):38–48. 23118818 PMC3484795

[40] Campbell M, Petti TA, Green WH, Cohen IL, Genieser NB, David R. Some Physical Parameters of Young Autistic Children. Journal of the American Academy of Child Psychiatry. 1980;19(2):193–212. 10.1016/S0002-7138(09)60697-X 7391427

[41] Elsheshtawy E, Tobar S, Sherra K, Atallah S, Elkasaby R. Study of some biomarkers in hair of children with autism:. Middle East Current Psychiatry. 2011;18(1):6–10. 10.1097/01.XME.0000392842.64112.64

[42] Priya MDL, Geetha A. Level of Trace Elements (Copper, Zinc, Magnesium and Selenium) and Toxic Elements (Lead and Mercury) in the Hair and Nail of Children with Autism. Biological Trace Element Research. 2011;142(2):148–158. 10.1007/s12011-010-8766-2 20625937

[43] Geier DA, Kern JK, King PG, Sykes LK, Geier MR. Hair Toxic Metal Concentrations and Autism Spectrum Disorder Severity in Young Children. International Journal of Environmental Research and Public Health. 2012;9(12):4486–4497. 10.3390/ijerph9124486 23222182 PMC3546773

[44] James SJ, Cutler P, Melnyk S, Jernigan S, Janak L, Gaylor DW, et al Metabolic biomarkers of increased oxidative stress and impaired methylation capacity in children with autism. The American Journal of Clinical Nutrition. 2004;80(6):1611–1617. 15585776 10.1093/ajcn/80.6.1611

[45] James SJ, Melnyk S, Jernigan S, Cleves MA, Halsted CH, Wong DH, et al Metabolic endophenotype and related genotypes are associated with oxidative stress in children with autism. American Journal of Medical Genetics Part B: Neuropsychiatric Genetics. 2006;141B(8):947–956. 10.1002/ajmg.b.30366 16917939 PMC2610366

[46] James SJ, Melnyk S, Fuchs G, Reid T, Jernigan S, Pavliv O, et al Efficacy of methylcobalamin and folinic acid treatment on glutathione redox status in children with autism. The American Journal of Clinical Nutrition. 2009;89(1):425–430. 10.3945/ajcn.2008.26615 19056591 PMC2647708

[47] Adams JB, Audhya T, McDonough-Means S, Rubin RA, Quig D, Geis E, et al Nutritional and metabolic status of children with autism vs. neurotypical children, and the association with autism severity. Nutrition & Metabolism. 2011;8(1):34 10.1186/1743-7075-8-34 21651783 PMC3135510

[48] Konstantareas MM, Homatidis S. Ear infections in autistic and normal children. Journal of Autism and Developmental Disorders. 1987;17(4):585–594. 3680158 10.1007/BF01486973

[49] Adams JB, Holloway CE, George F, Quig D. Analyses of toxic metals and essential minerals in the hair of Arizona children with autism and associated conditions, and their mothers. Biological Trace Element Research. 2006;110(3):193–209. 10.1385/BTER:110:3:193 16845157

[50] Adams JB, Romdalvik J, Ramanujam VMS, Legator MS. Mercury, Lead, and Zinc in Baby Teeth of Children with Autism Versus Controls. Journal of Toxicology and Environmental Health, Part A. 2007;70(12):1046–1051. 10.1080/15287390601172080 17497416

[51] Niehus R, Lord C. Early Medical History of Children with Autism Spectrum Disorders. Journal of Developmental & Behavioral Pediatrics. 2006;27(2):S120–S127. 10.1097/00004703-200604002-00010 16685178

[52] Rowland IR, Davies MJ, Evans JG. Tissue Content of Mercury in Rats Given Methylmercuric Chloride Orally: Influence of Intestinal Flora. Archives of Environmental Health: An International Journal. 1980;35(3):155–160. 10.1080/00039896.1980.10667485 7387196

[53] Rowland IR, Robinson RD, Doherty RA. Effects of Diet on Mercury Metabolism and Excretion in Mice Given Methylmercury: Role of Gut Flora. Archives of Environmental Health: An International Journal. 1984;39(6):401–408. 10.1080/00039896.1984.10545872 6524959

[54] Lord C, Rutter M, Goode S, Heemsbergen J, Jordan H, Mawhood L, et al Austism diagnostic observation schedule: A standardized observation of communicative and social behavior. Journal of Autism and Developmental Disorders. 1989;19(2):185–212. 10.1007/BF02211841 2745388

[55] Schopler E, Van Bourgondien M, Wellman J, Love S. Childhood Autism Rating Scale—Second edition (CARS2): Manual. Los Angeles: Western Psychological Services 2010;.

[56] Aman MG, Singh NN, Stewart AW, Field CJ. Psychometric characteristics of the aberrant behavior checklist. American Journal of Mental Deficiency. 1985;89(5):492–502. 3158201

[57] Rimland B, Edelson S. Autism treatment evaluation checklist: statistical analyses. Autism Research Institute 2000;.

[58] Constantino JN, Davis SA, Todd RD, Schindler MK, Gross MM, Brophy SL, et al Validation of a Brief Quantitative Measure of Autistic Traits: Comparison of the Social Responsiveness Scale with the Autism Diagnostic Interview-Revised. Journal of Autism and Developmental Disorders. 2003;33(4):427–433. 12959421 10.1023/a:1025014929212

[59] McIntosh DN, Miller LJ. Development and validation of the Short Sensory Profile In: Dunn W, editor. Sensory Profile Manual. Psychological Corporation; 1999 p. 59–73.

[60] Fisher RA. The Use of Multiple Measurements in Taxonomic Problems. Annals of Eugenics. 1936;7(2):179–188. 10.1111/j.1469-1809.1936.tb02137.x

[61] Mika S, R atsch G, Weston J, Sch olkopf B, M uller KR. Fisher Discriminant Analysis with Kernels. In: Proceedings of the Neural Networks for Signal Processing IX Workshop; 1999. p. 41–48.

[62] Li Y, Gong S, Liddell H. Recognising trajectories of facial identities using kernel discriminant analysis. Image and Vision Computing. 2003;21(13–14):1077–1086. 10.1016/j.imavis.2003.08.010

[63] Mardia KV, Kent JT, Bibby JM. Multivariate Analysis. Academic Press; 1980.

[64] Joliffe IT. Principal Component Analysis and Factor Analysis In: Principal Component Analysis. Springer Series in Statistics. Springer New York; 2002 p. 150–166. 10.1007/978-1-4757-1904-8_7

[65] Jackson JE. A User’s Guide to Principal Components. John Wiley & Sons; 2003.

[66] Kruger U, Xie L. Advances in statistical monitoring of complex multivariate processes: with applications in industrial process control. John Wiley & Sons; 2012.

[67] H oskuldsson A. PLS regression methods. Journal of Chemometrics. 1988;2(3):211–228.

[68] Burnham AJ, Viveros R, MacGregor JF. Frameworks for latent variable multivariate regression. Journal of Chemometrics. 1996;10(1):31–45. 10.1002/(SICI)1099-128X(199601)10:1<31::AID-CEM398>3.0.CO;2-1

[69] Burnham AJ, MacGregor JF, Viveros R. Latent variable multivariate regression modeling. Chemometrics and Intelligent Laboratory Systems. 1999;48(2):167–180. 10.1016/S0169-7439(99)00018-0

[70] Efron B. Estimating the Error Rate of a Prediction Rule: Improvement on Cross-Validation. Journal of the American Statistical Association. 1983;78(382):316–331.

[71] Steyerberg EW, Bleeker SE, Moll HA, Grobbee DE, Moons KGM. Internal and external validation of predictive models: A simulation study of bias and precision in small samples. Journal of Clinical Epidemiology. 2003;56(5):441–447. 10.1016/S0895-4356(03)00047-7 12812818

[72] Stone M. Cross-Validatory Choice and Assessment of Statistical Predictions. Journal of the Royal Statistical Society Series B (Methodological). 1974;36(2):111–147.

[73] Kohavi R. A Study of Cross-Validation and Bootstrap for Accuracy Estimation and Model Selection. In: Proceedings of the International Joint Conference on Artificial Intellegence; 1995. p. 1137–1143.

[74] Silverman BW. Density Estimation for Statistics and Data Analysis. CRC Press; 1986 10.1007/978-1-4899-3324-9

[75] Scott DW. Multivariate Density Estimation: Theory, Practice, and Visualization. John Wiley & Sons; 2015.

[76] Chen Q, Kruger U, Leung AT. Regularised kernel density estimation for clustered process data. Control Engineering Practice. 2004;12(3):267–274. 10.1016/S0967-0661(03)00083-2

[77] Blaurock-Busch E, Amin OR, Rabah T. Heavy Metals and Trace Elements in Hair and Urine of a Sample of Arab Children with Autistic Spectrum Disorder. Maedica. 2011;6(4):247–257. 22879836 PMC3391939

[78] Wright B, Pearce H, Allgar V, Miles J, Whitton C, Leon I, et al A Comparison of Urinary Mercury between Children with Autism Spectrum Disorders and Control Children. PLoS ONE. 2012;7(2):1–6. 10.1371/journal.pone.0029547 22355303 PMC3280241

[79] Albizzati A, Moré L, Di Candia D, Saccani M, Lenti C. Normal concentrations of heavy metals in autistic spectrum disorders. Minerva pediatrica. 2012;64(1):27–31. 22350041

[80] Hallmayer J, Cleveland S, Torres A, Phillips J, Cohen B, Torigoe T, et al Genetic heritability and shared environmental factors among twin pairs with autism. Archives of General Psychiatry. 2011;68(11):1095–1102. 10.1001/archgenpsychiatry.2011.76 21727249 PMC4440679

