# Supplementary material for: Significant Association of Urinary Toxic Metals and Autism-Related Symptoms—A Nonlinear Statistical Analysis with Cross Validation
Source: PLoS One. 2017 Jan 9;12(1):e0169526. doi: 10.1371/journal.pone.0169526 (PMC5222512; doi:10.1371/journal.pone.0169526)
Supplement: S1 Appendix — (PDF) [file pone.0169526.s001.pdf]

Defining the concentrations of toxic metals taken from the participants on the autism spectrum and the neurotypical participants by the sets $\mathcal{X}_1 = \{x_1(1), x_1(2), \ldots, x_1(n_1)\}$ and $\mathcal{X}_2 = \{x_2(1), x_2(2), \ldots, x_2(n_2)\}$, the mean vectors for both groups are $\bar{x}_1 = \frac{1}{n_1}\sum_{i=1}^{n_1} x_1(i)$ and $\bar{x}_2 = \frac{1}{n_2}\sum_{i=1}^{n_2} x_2(i)$, respectively. The sets $\mathcal{X}_1$ and $\mathcal{X}_2$ have $n_1 = 67$ and $n_2 = 50$ samples and each sample contains the corresponding measurements of the toxic metals for a particular participant. This allows defining the between cluster variation to be defined by the matrix $S_B = (\bar{x}_1 - \bar{x}_2)(\bar{x}_1 - \bar{x}_2)^{\mathrm{T}}$, which has the rank one. Next, the matrices $S_{x_1} = \sum_{i=1}^{n_1}(x_1(i) - \bar{x}_1)(x_1(i) - \bar{x}_1)^{\mathrm{T}}$ and $S_{x_2} = \sum_{i=1}^{n_2}(x_2(i) - \bar{x}_2)(x_2(i) - \bar{x}_2)^{\mathrm{T}}$ describe the variation of the samples of the participants on the autism spectrum and the neurotypical participants, respectively. The within variation of both sets, $\mathcal{X}_1$ and $\mathcal{X}_2$, is, consequently, $S_W = S_{x_1} + S_{x_2}$. The objective function for linear FDA seeks to maximize the ratio of the between over the within variation of both clusters, i.e. $J(w) = w^{\mathrm{T}} S_B w / w^{\mathrm{T}} S_W w$. The solution is the eigenvector of the matrix $S_W^{-1} S_B$. Note that the rank of this matrix is one.
