# Supplementary material for: Significant Association of Urinary Toxic Metals and Autism-Related Symptoms—A Nonlinear Statistical Analysis with Cross Validation
Source: PLoS One. 2017 Jan 9;12(1):e0169526. doi: 10.1371/journal.pone.0169526 (PMC5222512; doi:10.1371/journal.pone.0169526)
Supplement: S2 Appendix — (PDF) [file pone.0169526.s002.pdf]

KFDA carries out a nonlinear transformation of the random vector x into the feature space, producing $f = \psi(x)$, where the dimension of f is usually much higher than that of x. Instead of constructing the matrices S_B and S_W from the original random vector x, KFDA relies on the nonlinearly transformed vector f. The corresponding matrices describing the between and within variation of $\mathcal{F}_1 = \{f_1(1) = \psi(x_1(1)), f_1(2) = \psi(x_1(2)), \dots, f_1(n_1) = \psi(x_1(n_1))\}$ and $\mathcal{F}_2 = \{f_2(1) = \psi(x_2(1)), f_2(2) = \psi(x_2(2)), \dots, f_2(n_2) = \psi(x_2(n_2))\}$ are $S_B^f = (\bar{f}_1 - \bar{f}_2)(\bar{f}_1 - \bar{f}_2)^{\mathrm{T}}$ and $S_W^f = S_{f_1} + S_{f_2}$. Here, $\bar{f}_1 = \frac{1}{n_1}\sum_{i=1}^{n_1}\psi(x_1(i))$, $\bar{f}_2 = \frac{1}{n_2}\sum_{i=1}^{n_2}\psi(x_2(i))$, $S_{f_1} = \sum_{i=1}^{n_1}(\psi(x_1(i)) - \bar{f}_1)(\psi(x_1(i)) - \bar{f}_1)^{\mathrm{T}}$ and $S_(f_2) = \sum_{i=1}^{n_2}(\psi(x_2(i)) - \bar{f}_2)(\psi(x)_2(i)) - \bar{f}_2)^{\mathrm{T}}$. The nonlinear transformation $f = \psi(x)$ is usually not known, however, [61] showed how to utilize scalar products $\psi(x(i)) \cdot \psi(x(j))$, which can be expressed by Mercer kernels $k(x(i), x(j)) = \psi(x(i)) \cdot \psi(x(j))$. Mercer kernels include Gaussian RBF, $k(x(i), x(j)) = exp(\|x(i) - x(j)\|/c)$, or polynomial kernels, $k(x(i), x(j)) = (x(i) \cdot x(j))^d$, where $\| \cdot \|$ is the squared norm of a vector, and c and d are positive parameters. In this work, we used Gaussian RBF kernels and selected the parameter c using cross validation, as advocated by [81] for kernel based classification problems.

Following from the theory of reproducing kernels, the solution for the objective function $J(w) = (w^{\mathrm{T}} S_B^f w)/(w^{\mathrm{T}} S_W^f w)$ can be expressed as a function of the nonlinearly transformed samples, i.e. $w = \sum_{i=1}^{n}\alpha_i\phi(x(i))$. This implies that $w^{\mathrm{T}}\bar{f}_1$ can be written as $\frac{1}{n_1}\sum_{i=1}^{n}\alpha_i\sum_{j}^{n_1}k(x(i), x_1(j))$, where the ith element is $\alpha_i \cdot \frac{1}{n_1}\sum_{j=1}^{n_1}k(x(i), x_1(j)) = \alpha_i m_{1i}$. Expressing the sums $w^{\mathrm{T}}\bar{f}_1 = \sum_{i=1}^{n}\alpha_i m_{1i}$ and $w^{\mathrm{T}}\bar{f}_2 = \frac{1}{n_2}\sum_{i=1}^{n}\alpha_i\sum_{j=1}^{n_2}k(x(i), x_2(j)) = \sum_{i=1}^{n}\alpha_i m_{2i}$ as a scalar product yields $\alpha^{\mathrm{T}} m_1$ and $\alpha^{\mathrm{T}} m_2$, so that the numerator of the objective function $J(w)$, $w^{\mathrm{T}} S_B^f w$, becomes $\alpha^{\mathrm{T}} M\alpha$, where $M = (m_1 - m_2)(m_1 - m_2)^{\mathrm{T}}$. Analogously, the denominator of $J(w)$, $w^{\mathrm{T}} S_W^f w$ can be written as $\alpha^{\mathrm{T}} N\alpha$. The construction of N follows from the definition of $w^{\mathrm{T}} S_{f_1} w$ and $w^{\mathrm{T}} S_{f_2} w$. Substituting $w = \sum_{i=1}^{n}\alpha_i\psi(x(i))$ into $w^{\mathrm{T}} S_{f_1} w$ gives rise to $\alpha^{\mathrm{T}} K_1[I_{n_1} - 1_{n_1}]K_1^{\mathrm{T}}\alpha$, where K_1 is a matrix of dimension n times n_1 for which $k(x(i), x_1(j))$ is the element in the ith row and jth column, I_{n_1} is the identify matrix of dimension n_1 and 1_{n_1} is a matrix for which each element is $1/n_1$. Analogously, $w^{\mathrm{T}} S_{f_2} w = \alpha^{\mathrm{T}} K_2[I_{n_2} - 1_{n_2}]K_2^{\mathrm{T}}\alpha$, where K_2 is a n by n_2 matrix that has $k(x(i), x_2(j))$ as the element in the ith row and jth column. The matrices I_{n_2} and 1_{n_2} are the identify matrix of dimension n_2 and a matrix for which every entry is $1/n_2$, respectively. Hence, $N = K_1[I_{n_1} - 1_{n_1}]K_1^{\mathrm{T}} + K_2[I_{n_2} - 1_{n_2}]K_2^{\mathrm{T}}$. The optimal solution for α is given by the eigenvector of the matrix $N^{-1}M$. Caution is required here, since the rank of N is less than n [61]. This can be overcome by utilizing a regularization parameter of the generalized inverse. The results presented in this article relied on the use of the generalized inverse.

References

[61] Hsu CW, Chang CC, Lin CJ. A practical guide to support vector classification. Department of Computer Science, National Taiwan University; 2003. Available from: `http://www.csie.ntu.edu.tw/ cjlin/papers/guide/guide.pdf`

[81] Mika S, R atsch G, Weston J, Sch olkopf B, M uller KR. Fisher Discriminant Analysis with Kernels. In: Proceedings of the Neural Networks for Signal Processing IX Workshop; 1999. p. 41–48
