# Supplementary material for: Significant Association of Urinary Toxic Metals and Autism-Related Symptoms—A Nonlinear Statistical Analysis with Cross Validation
Source: PLoS One. 2017 Jan 9;12(1):e0169526. doi: 10.1371/journal.pone.0169526 (PMC5222512; doi:10.1371/journal.pone.0169526)
Supplement: S3 Appendix — (PDF) [file pone.0169526.s003.pdf]

This multivariate technique determines linear combinations of an m-variate set of random variables that possess a mean of zero that has a maximum variance, i.e. $t_j = p_{1j}x_1 + p_{2j}x_2 + \cdots + p_{ij}x_i + \cdots + p_{mj}x_m = \sum_{i=1}^{m} p_{ij}x_i = p_j^{\mathrm{T}} x$ and $E\{t_j^2\}$ is a maximum. By setting the vector p_j to be of unit length, the PCA objective function is $J(p_j) = E\{p_j^{\mathrm{T}} x x^{\mathrm{T}} p_j\} - \lambda_j (p_j^{\mathrm{T}} p_j - 1)$ (Kruger and Xie, 2012). As $E\{p_j^{\mathrm{T}} x x^{\mathrm{T}} p_j\} = p_j^{\mathrm{T}} E\{x x^{\mathrm{T}}\} p_j = p_j^{\mathrm{T}} S_{xx} p_j$, where S_{xx} is the covariance matrix of the random vector x, the maximum of $J(p_j)$ is given by the eigenvector associated with the jth largest eigenvector of S_{xx} [66]. The orthogonal projection of a sample onto the direction vector p_j is given by $(p_j^{\mathrm{T}} x)p_j$ and the orthogonal distance of the projection and the sample is $x - (p_j^{\mathrm{T}} x)p_j$. The variance of t_j is equal to the jth largest eigenvalue of S_{xx}, such that the variance of t_1 is larger or equal to that of t_2. Generally, $E = \{t_j^2\} \leq E\{t_{j+1}^2\}$. Consequently, the eigenvalue plot reveals how many important principal components the variable set x contains. Moreover, the linear combinations of the dominant principal components reveal variable interrelationships among the variable set in x. For example, plotting the elements of p_1 versus the elements of p_2, i.e. the two most important principal components, to produce a scatter diagram yields, graphically, the most dominant variable interrelationships in form of clusters.

References

[66] Kruger U, Xie L. Advances in statistical monitoring of complex multivariate processes: with applications in industrial process control. John Wiley & Sons; 2012
