# Supplementary material for: Significant Association of Urinary Toxic Metals and Autism-Related Symptoms—A Nonlinear Statistical Analysis with Cross Validation
Source: PLoS One. 2017 Jan 9;12(1):e0169526. doi: 10.1371/journal.pone.0169526 (PMC5222512; doi:10.1371/journal.pone.0169526)
Supplement: S4 Appendix — (PDF) [file pone.0169526.s004.pdf]

The objective function for determining the weight vectors for $x$ and $y$ is the covariance criterion $E\{(w^{\mathrm{T}}x)(v^{\mathrm{T}}y)\}$, where $E\{\cdot\}$ is the expectation operator. Denoting $t = w^{\mathrm{T}}x$ and $u = v^{\mathrm{T}}y$ as score variables, the covariance criterion between $t$ and $u$ becomes $E\{tu\}$, where the weight vectors are constraint to be of unit length [67]. Prior to the determination of subsequent pairs of weight vectors, the information that is encapsulated in $t$ is subtracted, or deflated, from $x$ and $y$. This yields the deflation procedure $x = xtp^{\mathrm{T}}$ and $y = ytq^{\mathrm{T}}$, where the loading vectors are $p = E\{xt\}/E\{t^2\}$ and $q = E\{yt\}/E\{t^2\}$. Stacking the weight and loading vectors as columns to form the matrices $W = \begin{bmatrix} w_1 & w_2 & \dots & w_n \end{bmatrix}$, $V = \begin{bmatrix} v_1 & v_2 & \dots & v_n \end{bmatrix}$, $P = \begin{bmatrix} p_1 & p_2 & \dots & p_n \end{bmatrix}$ and $Q = \begin{bmatrix} q_1 & q_2 & \dots & qn \end{bmatrix}$, the parametric regression matrix is given by $B = Q[W^{\mathrm{T}}P]^{-1}W^{\mathrm{T}}$. Note that $R^{\mathrm{T}} = [W^{\mathrm{T}}P]^{-1}W^{\mathrm{T}}$ can be directly determined from the PLS algorithm [66, 67] and hence $B = QR^{\mathrm{T}}$.

# References


[66] Kruger U, Xie L. Advances in statistical monitoring of complex multivariate processes: with applications in industrial process control. John Wiley & Sons; 2012

[67] H oskuldsson A. PLS regression methods. Journal of Chemometrics. 1988;2(3):211–228
