# Supplementary material for: Significant Association of Urinary Toxic Metals and Autism-Related Symptoms—A Nonlinear Statistical Analysis with Cross Validation
Source: PLoS One. 2017 Jan 9;12(1):e0169526. doi: 10.1371/journal.pone.0169526 (PMC5222512; doi:10.1371/journal.pone.0169526)
Supplement: S5 Appendix — (PDF) [file pone.0169526.s005.pdf]

To remove the limitation of determining a linear parametric regression model between x and y, generic nonlinear extensions to PLS are based on reproducing kernels [82, 83]. The application of this kernel PLS or KPLS algorithm commences by defining a nonlinear transformation of the predictor variable set x, that is $f = \psi(x)$ in a similar fashion to that discussed for KFDA. Typically, the dimension of f is significantly larger than that of x and the corresponding space for f is referred to as the feature space. Instead of regressing x on y, KPLS regresses the transformed variable set f on y using a modified version of the linear PLS algorithm [82, 83].

The modified PLS algorithm in the feature space relies on the matrix product XX^{T}, $X^{\mathrm{T}} = \begin{bmatrix} x_1 & x_2 & \dots & x_n \end{bmatrix}$, which in the feature space becomes the symmetric and positive semidefinite kernel matrix $K(X, X) = \phi(X)\phi^{\mathrm{T}}(X)$, for which k_{ij} is the element in the ith row and the jth column. After removing the mean vector of $\phi(x)$, the centered Kernel or Gram matrix then forms the basis for determining the regression matrix. To derive this relationship, it is important to note that the linear PLS regression matrix can be computed as $B = Q[W^{\mathrm{T}}P]^{-1}W^{\mathrm{T}} = Y^{\mathrm{T}}T[U^{\mathrm{T}}XX^{\mathrm{T}}T]^{-1}U^{\mathrm{T}}X$, according to [84]. Here, the matrix $T = \begin{bmatrix} t_1 & t_2 & \dots & t_n \end{bmatrix}$ and $U = \begin{bmatrix} u_1 & u_2 & \dots & u_n \end{bmatrix}$ are matrices storing the values of the t- and u-score variables for each sample and each projection direction as column and row vectors, respectively. By replacing the data matrix X by $\phi(X)$ after mean centering, the nonlinear regression equation matrix becomes $B = Y^{\mathrm{T}}T[U^{\mathrm{T}}\phi(X)\phi^{\mathrm{T}}(X)^{\mathrm{T}}T]^{-1}U^{\mathrm{T}}\phi(X)$. The prediction of a new sample, $y \notin Y$ and $x \notin X$ is then $\hat{y} = Y^{\mathrm{T}}T[U^{\mathrm{T}}\phi(X)\phi^{\mathrm{T}}(X)^{\mathrm{T}}T]^{-1}U^{\mathrm{T}}\phi(X)\phi^{\mathrm{T}}(x)$ or $Y^{\mathrm{T}}T[U^{\mathrm{T}}K(X, X)^{\mathrm{T}}T]^{-1}U^{\mathrm{T}}k(X, x)$, where $k^{\mathrm{T}}(X, x)$ is a vector of the mean centered elements of the Gaussian kernel function.

References

[82] Rosipal R, Trejo LJ. Kernel Partial Least Squares Regression in Reproducing Kernel Hilbert Space. J Mach Learn Res. 2002;2:97–123

[83] Kim K, Lee JM, Lee IB. A novel multivariate regression approach based on kernel partial least squares with orthogonal signal correction. Chemometrics and Intelligent Laboratory Systems. 2005;79(12):22–30

[84] Rännar S, Lindgren F, Geladi P, Wold S. A PLS kernel algorithm for data sets with many variables and fewer objects. Part 1: Theory and algorithm. Journal of Chemometrics. 1994;8(2):111–125
