# Supplementary material for: Significant Association of Urinary Toxic Metals and Autism-Related Symptoms—A Nonlinear Statistical Analysis with Cross Validation
Source: PLoS One. 2017 Jan 9;12(1):e0169526. doi: 10.1371/journal.pone.0169526 (PMC5222512; doi:10.1371/journal.pone.0169526)
Supplement: S1 Table — (PDF) [file pone.0169526.s006.pdf]

Subject Number	Al /creat	As /creat	Cd /creat	Cs /creat	Hg /creat	Ni /creat	Pb /creat	Sn /creat	Tl /creat	W /creat
1	13.19679	4.78486	0.12355	5.21273	0.78489	6.37844	0.99475	0.13	0.12202	0.39809
2	3.33	7.63152	0.24879	1.82148	0.76162	2.76005	0.46474	0.13	0.08758	0.0968
3	10.89028	4.77429	0.24973	1.66671	0.38501	2.73905	0.73482	0.38668	0.08129	0.13935
4	3.33	2.31675	0.19344	3.38477	0.65842	3.65028	0.46013	0.88147	0.15104	0.07802
5	10.48063	17.26349	0.80469	5.60097	0.48972	7.49344	0.89523	4.29568	0.17206	1.15805
6	5.12932	5.56302	0.33382	4.18856	0.60109	3.19878	0.70231	0.44903	0.39161	0.10961
7	9.13201	5.00113	0.4435	4.16755	0.36131	4.52866	0.26847	1.18127	0.16268	0.41331
8	3.33	5.34524	0.20621	1.27321	0.83709	1.48282	0.23311	0.22509	0.04481	0.02668
9	10.46603	10.17315	0.48591	2.05066	0.45747	1.35797	0.4647	2.36852	0.14194	0.33281
10	7.25302	13.02085	0.27607	4.47877	0.53284	2.66638	1.15949	0.76825	0.28745	0.1928
11	3.33	23.04456	0.30588	6.72478	1.88431	1.36684	0.64777	2.28734	0.1713	0.38146
12	3.33	24.97253	0.42641	3.33887	0.40837	1.9529	0.47492	0.26787	0.04725	0.23062
13	16.01853	3.59204	0.47012	4.98361	2.96387	3.68692	0.66336	13.90063	0.16995	0.95225
14	9.5341	7.84672	0.45738	2.83377	0.2	7.47787	0.56475	2.3761	0.08459	0.11934
15	3.33	9.83098	0.3991	5.09678	0.2	2.54172	0.94215	2.375	0.08627	0.19731
16	5.07181	3.60778	0.3277	2.65723	0.51507	3.7644	0.20207	0.40851	0.1158	0.08809
17	3.33	7.3417	0.30084	5.04519	0.2	2.0788	2.52455	12.67852	0.25307	0.42552
18	3.33	8.95685	0.62255	7.34768	0.2	3.70065	0.45653	6.66041	0.16827	0.56214
19	13.41954	4.08306	0.24787	4.48787	0.2	2.45296	0.59704	1.13001	0.225	0.36278
20	6.92319	2.99312	0.16474	2.36061	0.2	2.85951	0.69869	4.02111	0.13548	0.24327
21	7.36005	12.15699	0.66667	9.24327	0.69675	7.47748	0.75013	3.64723	0.56666	0.77186
22	8.09959	10.13449	0.55783	7.46506	1.84623	9.73479	1.49096	1.02831	0.33493	1.20407
23	3.33	3.24028	0.30989	3.92111	0.34691	5.64991	0.59355	0.63754	0.18891	0.14108
24	6.08696	24.28656	0.24177	2.05986	0.2	2.07524	0.24947	23.90098	0.11397	0.10215
25	3.33	129.08127	0.3017	5.9177	1.07455	3.46063	0.68428	1.49014	0.20887	0.28779
26	10.19663	4.04628	0.33107	1.66033	3.22314	0.46017	0.52959	5.07355	0.15184	0.17769
27	3.33	13.08541	0.27223	3.67772	0.50642	3.83583	0.07	0.72226	0.20624	0.14398
28	3.33	9.11209	0.18916	2.51147	0.75153	2.47142	0.2269	0.13	0.15666	0.04843
29	5.56416	43.92906	0.36034	2.9984	0.2	1.98522	2.23058	2.52605	0.11985	0.30706
30	10.28742	11.27244	0.15945	5.15946	0.31176	4.72155	0.32102	2.88113	0.16765	0.29811
31	20.80033	18.75139	0.24152	7.87781	0.2	4.11786	0.38426	1.74494	0.19299	0.19967
32	37.49616	7.72054	0.26032	5.2376	0.2	12.95556	0.54781	3.6828	0.14739	0.277
33	12.09373	11.392	0.67885	2.2094	0.7926	5.93792	0.54723	1.92574	0.09798	0.47227
34	3.33	3.6359	0.24874	1.45445	1.48074	1.6227	0.1342	0.37843	0.10586	0.11944
35	11.91161	13.41525	0.28786	4.0434	0.2	8.50046	0.77678	2.07747	0.19431	0.1286
36	3.33	26.08864	0.16904	4.79614	1.65947	2.16	0.48409	0.72388	0.13246	0.09594
37	3.33	27.64852	0.18456	5.71	0.2	3.08851	0.9904	0.4045	0.14129	0.28905
38	3.33	1.58972	0.18335	2.22208	0.2	5.95336	0.37591	0.27455	0.08249	0.21102
39	7.08954	18.76952	0.44194	5.06952	1.39644	2.3079	0.07	2.14961	0.15895	0.29522
40	6.76357	44.66544	1.03562	4.43733	0.71125	5.92258	0.17946	2.29283	0.10676	0.45664
41	9.79115	11.69827	0.27652	3.83898	0.43116	4.51371	0.37794	0.70677	0.10008	0.21663
42	24.30904	60.5698	0.46903	5.23814	0.42295	5.6802	0.5349	2.88208	0.19445	0.27833
43	12.53653	32.34857	0.23768	4.31146	0.73711	1.29284	1.48653	0.2851	0.32951	0.6414
44	3.33	14.04751	0.09953	2.02907	0.2	6.76547	0.2952	1.70323	0.19211	0.04877
45	8.24037	5.9345	0.61729	6.05522	0.5278	4.32224	0.81478	5.39094	0.24966	0.43846
46	6.39556	9.89234	0.29296	4.12726	0.83277	4.02159	0.19893	0.95942	0.28147	0.19112
47	7.47065	4.03239	0.18	2.17885	0.44201	3.65101	0.29266	0.13	0.07683	0.16532
48	13.28498	10.8764	0.46089	4.26936	0.2	7.63465	0.79604	3.12607	0.12541	0.46997
49	11.55709	4.07206	0.18503	2.68141	0.46653	5.96784	0.29407	0.37497	0.06111	0.13668
50	10.70659	20.62855	0.22203	5.16912	0.2	5.50147	0.46199	1.13033	0.22227	0.21877
51	16.53497	6.57711	0.29419	5.27111	0.2	3.54262	0.52105	0.8222	0.16788	0.13292
52	11.12301	14.38312	0.65028	5.22801	0.2	7.58757	0.82245	6.33525	0.20594	0.30223
53	12.1694	9.32259	0.37813	3.17864	0.2	4.33645	0.39651	3.81273	0.12933	0.18439
54	5.67307	6.64386	0.31585	3.3487	0.2	4.03962	1.52799	19.22979	0.23553	0.11087
55	3.33	2.6649	0.16023	2.81313	0.2	2.45972	0.11705	0.34735	0.08965	0.19343
56	3.33	4.6374	0.16648	1.58772	0.2	2.002	0.1128	0.13	0.05706	0.25062
57	3.33	2.88457	0.18469	1.16382	0.2	2.12867	0.20591	0.37133	0.04257	0.15245
58	3.33	5.62183	0.28987	3.79325	0.2	2.98204	0.22175	1.08015	0.10807	0.26046
59	9.49883	8.38934	0.48176	10.41932	0.2	5.14745	0.87573	4.24451	0.31877	1.05394
60	5.96244	4.9109	0.30133	2.13976	0.41119	3.99323	0.19219	0.20545	0.11973	0.19234
61	29.63626	5.83502	0.17487	5.29568	0.2	6.13973	0.37208	0.37871	0.20323	0.41849
62	15.36425	5.42324	0.28976	2.79887	1.17033	9.96135	0.33793	13.2538	0.07014	0.1493
63	7.00373	12.38097	0.22127	4.79496	0.3209	7.30299	0.25336	0.24067	0.15709	0.13377
64	10.4905	9.51966	0.49998	3.93612	2.05195	7.20344	1.12249	0.78833	0.44403	0.04521
65	20.96215	4.03181	0.09124	1.67869	0.2	1.47052	0.1709	0.13	0.06281	0.06583
66	13.31799	7.97443	0.24589	1.4741	0.2	2.91758	0.17757	0.13	0.06323	0.19643
67	11.54239	16.87341	0.48179	4.90539	2.29604	4.77995	0.97872	0.69723	0.19253	0.27457
