# Supplementary material for: Significant Association of Urinary Toxic Metals and Autism-Related Symptoms—A Nonlinear Statistical Analysis with Cross Validation
Source: PLoS One. 2017 Jan 9;12(1):e0169526. doi: 10.1371/journal.pone.0169526 (PMC5222512; doi:10.1371/journal.pone.0169526)
Supplement: S2 Table — (PDF) [file pone.0169526.s007.pdf]

Subject Number	Al /creat	As /creat	Cd /creat	Cs /creat	Hg /creat	Ni /creat	Pb /creat	Sn /creat	Tl /creat	W /creat
68	3.33	8.04837	0.17902	2.02038	0.2	3.63181	0.42196	0.22433	0.07964	0.30482
69	3.33	5.49932	0.23322	1.95517	1.014	2.29856	0.41568	0.13	0.09593	0.21992
70	3.33	8.51679	0.26636	3.10691	0.2	2.56239	0.29968	0.13	0.07149	0.10691
71	12.08868	13.71827	0.1979	2.50344	0.2	4.76587	0.32227	1.32299	0.10922	0.2485
72	6.42861	4.88458	0.20641	3.12727	0.33335	2.488	0.70752	0.13	0.08484	0.12829
73	3.33	1.38114	0.09522	1.47754	0.2	3.95718	0.5915	0.13	0.04526	0.30804
74	3.33	2.27673	0.36036	3.11622	0.31349	2.08307	0.32575	0.51209	0.22664	0.06956
75	11.08885	41.44383	0.20781	3.63525	0.6397	3.16908	0.42926	1.53052	0.08327	0.28922
76	7.0412	12.7652	0.63923	4.83211	0.3125	4.60703	0.36955	2.54167	0.07048	0.28873
77	10.96169	36.93132	0.52179	5.88766	0.59178	3.00243	0.70845	4.2646	0.09912	0.45079
78	10.79133	12.62281	0.30348	1.93421	0.2	3.30617	0.56174	0.59126	0.09042	0.51518
79	3.33	2.22006	0.12423	1.13438	0.2	1.24958	0.09549	0.13	0.05141	0.12401
80	6.96546	5.22715	0.24515	1.83732	0.2	2.66082	0.28072	0.24515	0.08155	0.24567
81	9.24671	16.36943	0.56846	3.90518	0.2	3.58428	0.18135	0.38955	0.1066	0.23408
82	3.33	11.06547	0.52801	4.7105	0.2	4.32964	0.32679	0.24853	0.068	0.26767
83	15.91917	7.23759	0.26033	3.3283	0.2	3.35826	0.47975	2.43726	0.07311	0.2912
84	7.48232	11.39087	0.43612	2.8673	0.35513	2.00551	0.07	1.2154	0.08859	0.27947
85	7.60758	8.98194	0.32467	6.06432	0.45419	6.00991	0.27665	0.76586	0.14145	0.51795
86	3.33	8.48717	0.20156	4.86335	0.2	3.59815	0.24421	0.54696	0.10909	0.18272
87	3.33	3.85425	0.18006	3.50655	0.2	8.53729	0.07	0.13	0.13578	0.16494
88	8.91813	11.99916	0.29275	6.98908	0.2	4.48855	0.61124	1.81912	0.2867	0.19611
89	3.33	4.00764	0.31495	2.70181	0.2	2.48562	0.3105	1.68416	0.16489	0.15214
90	3.33	1.99343	0.41443	2.58148	0.30382	1.94076	0.21322	0.74755	0.09099	1.16056
91	3.33	12.09917	0.24903	2.51246	0.83834	3.46447	0.22132	0.34576	0.0664	0.04034
92	3.33	5.19225	0.15794	1.88246	0.62988	4.54255	0.26418	0.40465	0.0568	0.12502
93	3.33	7.39322	0.45776	3.13097	0.56454	1.60474	0.2924	4.38105	0.11165	0.26837
94	3.33	6.05954	0.11934	1.64779	0.2	3.68458	0.12504	0.13	0.06928	0.06428
95	3.33	7.27671	0.50497	2.62081	1.39301	4.04317	0.31165	1.16894	0.06137	0.45776
96	24.73399	7.80689	0.42686	4.03358	0.98627	4.48936	0.66981	0.39907	0.08255	0.13801
97	5.45168	12.11831	0.34365	3.76205	0.75737	7.26069	0.30784	0.27369	0.08038	0.51737
98	43.52436	14.56682	0.62418	4.31827	0.12117	2.32452	0.20976	2.41971	0.10819	0.42403
99	3.33	4.94686	0.32626	3.34857	0.96043	1.82063	0.21355	0.42634	0.06694	0.08602
100	3.33	4.2268	0.21112	1.17669	0.45561	3.93719	0.11031	0.71323	0.07713	0.29347
101	3.33	18.85891	0.18542	3.20132	0.2	1.39231	0.16261	0.66018	0.17462	0.19679
102	7.58982	10.21478	0.23801	3.42515	0.89591	2.65849	0.28085	0.80991	0.0488	0.26925
103	13.96414	7.95378	0.25142	4.97507	0.61347	4.87498	0.22982	0.57575	0.15255	0.12612
104	13.45164	13.4527	0.37653	10.63345	0.2	3.89777	0.57218	2.87911	0.22535	0.32242
105	5.15714	8.3462	0.22411	7.23678	0.2	1.69108	0.21702	0.62817	0.23364	0.58946
106	3.33	11.21572	0.38286	2.43665	0.2	4.61295	0.24155	2.69826	0.07622	0.12496
107	5.73899	15.91424	1.79376	5.93395	1.06156	7.21807	0.57404	0.90318	0.16232	0.31073
108	12.62186	24.32236	0.88755	4.85934	0.56696	12.33914	0.60826	1.72465	0.12058	0.25921
109	3.33	16.03183	1.1715	5.37535	0.39504	8.57298	0.48527	1.02361	0.09637	0.43516
110	6.80746	12.67064	0.80549	4.25689	0.2	3.12851	0.40598	1.0381	0.08906	1.02896
111	7.00324	9.2978	0.54	3.27364	3.84058	5.23214	0.24624	0.34208	0.17723	0.15364
112	5.7651	29.48961	0.49253	5.13384	0.41737	6.28449	0.59058	1.44665	0.11084	0.47148
113	3.33	9.05984	0.20171	3.81209	0.40419	6.85101	0.37023	0.54713	0.1276	0.10574
114	6.43692	12.84875	0.2821	5.07585	0.2	3.96882	0.51972	0.49192	0.1556	0.17819
115	8.26415	7.09744	0.21146	4.3566	0.35229	1.58679	0.29111	0.30243	0.18652	0.12978
116	69.95514	7.63931	0.30587	2.88124	2.42423	3.71868	0.12284	0.31215	0.07708	0.42887
117	3.33	21.13142	0.43429	3.68775	0.2	5.20119	0.33755	0.57709	0.09372	0.26412
