# Supplementary material for: Significant Association of Urinary Toxic Metals and Autism-Related Symptoms—A Nonlinear Statistical Analysis with Cross Validation
Source: PLoS One. 2017 Jan 9;12(1):e0169526. doi: 10.1371/journal.pone.0169526 (PMC5222512; doi:10.1371/journal.pone.0169526)
Supplement: S3 Table — (PDF) [file pone.0169526.s008.pdf]

Subject Number	Irritability	Lethargy	Stereotypy	Hyperactivity	Inappropriate Speech	ABC Total
1	36	40	14	38	5	133
2	7	9	2	6	2	26
3	7	20	3	13	0	43
4	8	10	11	27	12	68
5	16	21	5	27	5	74
6	6	6	3	17	5	37
7	12	16	5	25	9	67
8	10	32	12	12	7	73
9	2	0	2	9	2	15
10	11	0	2	8	5	26
11	24	12	6	37	4	83
12	0	8	0	3	6	17
13	24	13	6	21	2	66
14	11	22	15	16	6	70
15	12	3	10	22	8	55
16	10	0	7	8	5	30
17	34	26	12	41	7	120
18	33	34	19	40	12	138
19	4	10	5	9	2	30
20	21	9	6	37	12	85
21	19	12	2	37	10	80
22	33	15	7	32	7	94
23	15	5	7	29	8.5	64.5
24	18	14	3	35	2	72
25	21	30	12	32	6	101
26	20	15	9	41	11	96
27	16	36	14	23	8	97
28	3	9	1	6	0	19
29	11	29	12	38	5	95
30	27	20	14	38	6	105
31	16	14	10	41	8	89
32	31	31	21	46	12	141
33	24	7	15	27	9	82
34	25	18	15	11	6	75
35	35	26	16	43	9	129
36	25	17	9	20	7	78
37	15	19	18	26	2	80
38	0	0	0	0	0	0
39	19	7	6	32	8	72
40	25	31	12	40	3	111
41	26	16	2	25	0	69
42	19	21	14	34	5	93
43	9	14	10	25	1	59
44	1	15	9	34	7	66
45	25	13	7	20	3	68
46	35	12	11	45	5	108
47	1	8	0	0	0	9
48	7	0	0	18	0	25
49	4	23	7	7	0	41
50	27	4	13	17	5	66
51	6	9	3	19	8	45
52	2	9	1	6	0	18
53	21	12	6	40	7	86
54	2	17	9	12	2	42
55	1	17	4	7	4	33
56	0	9	0	5	7	21
57	3	15	0	3	2	23
58	9	9	10	22	6	56
59	36	5	6	44	6	97
60	6	20	2	6	2	36
61	11	9	9	10	2	41
62	8	29	18	23	5	83
63	17	9	10	18	7	61
64	22	31	13	29	9	104
65	6	9	5	10	4	34
66	14	12	0	8	1	35
67	12	4	7	25	4	52
