# Supplementary material for: Significant Association of Urinary Toxic Metals and Autism-Related Symptoms—A Nonlinear Statistical Analysis with Cross Validation
Source: PLoS One. 2017 Jan 9;12(1):e0169526. doi: 10.1371/journal.pone.0169526 (PMC5222512; doi:10.1371/journal.pone.0169526)
Supplement: S4 Table — (PDF) [file pone.0169526.s009.pdf]

Subject Number	ABC Total	ATEC Total	PDDBI	PGIR2	SAS	SRS	SSP	CARS	Pro SAS	Raw ADOS	Adj ADOS
1	133	123	74	2.470588235	10	165	79	53	10	20	18
2	26	34	-155	0.882352941	6	87	154	29.5	4	9	9
3	43	47	-125	1.058823529	2.5	91	144	34.5	6	14	14
4	68	69	-74	2.117647059	5	110	70	38.5	7	25	22
5	74	96	-23	1.352941176	7	134	117	42	7	23	21
6	37	39	-119	0.941176471	4	109	134	39	7	17	16
7	67	71	-92	1.764705882	4	132	84	39.5	7	14	14
8	73	103	36	2	7	170	77	44.5	8	22	20
9	15	41	-123	1.117647059	6	57	127	37	8	16	16
10	26	19	-147	0.529411765	3	43	151	36	7	16	15
11	83	55	-71	1.941176471	1.5	106	123	35.5	4	2	2
12	17	58	-108	1.411764706	7	92	103	35	6	20	20
13	66	57	-118	1.529411765	4	119	128	42	7	10	10
14	70	88	34	1.764705882	6	154	108	40	8	13	13
15	55	63	-80	1.470588235	6	113	165	38	8	12	12
16	30	39	-76	1.588235294	3	103	104	35.5	7	9	9
17	120	102	-31	2	4.5	133	79	33	3	9	9
18	138	115	-36	1.941176471	4	144	67	27.5	3	8	8
19	30	63	-42	1.294117647	7	114	99	38.5	9	10	10
20	85	69	-71	1.764705882	7	121	121	41	8	17	17
21	80	69	-83	1.235294118	3	98	148	37.5	6	9	9
22	94	77	-24	1.705882353	5	78	116	40	7	19	17
23	64.5	73.5	-89	1.941176471	6	96	116	45	8	22	21
24	72	65	27	1.588235294	8	127	105	47.5	10	28	20
25	101	102	-52	1.588235294	4	149	138	40	7	13	13
26	96	89	-3	1.823529412	6	119	119	45.5	7	29	23
27	97	57	-153	1.058823529	2	82.5	118	37.5	7	12	12
28	19	15	-186	0.823529412	2	58	145	32.5	4	6	6
29	95	70	-54	1.6875	4	129	126	38	5	15	15
30	105	81	5	1.647058824	3	128	112	40.5	6	11	11
31	89	65	-39	1.235294118	4	146	97	41	7	16	16
32	141	124	21	2.294117647	4	161	73	39	6	15	15
33	82	82	-11	1.941176471	3	145	83	39.5	6	10	10
34	75	79	-9	2.235294118	5	134	110.4	49.5	8	22	21
35	129	103	-21	2.411764706	6	154	76	42.5	6	16	15
36	78	62	-80	1.529411765	4	125	97	52.5	8	18	18
37	80	100	4	1.823529412	8	139	101	51	9	19	18
38	0	5	-202	0.411764706	1	5	178	30	3	3	3
39	72	39	-114	1	3	116	123	32	6	6	6
40	111	102	30	1.882352941	8	142	120	49	9	26	20
41	69	53	-107	1.294117647	3	77	110	36.5	5	8	8
42	93	86	-7	2.235294118	10	133	109	44.5	9	15	14
43	59	93	3	1.529411765	3	120	109	38	8	18	18
44	66	85	-32	1.705882353	6	131	119	38.5	6	7	7
45	68	69	-26	1.882352941	7	113	122	46	8	18	17
46	108	139.5	65	2.941176471	10	146	79	34.5	3	20	13
47	9	20	-167	0.823529412	2	68	159	40	6	17	16
48	25	39	-124	0.941176471	2.5	42	126	33.5	4	8	8
49	41	45	-92	1	1	115	120	34.5	4	9	9
50	66	63	-81	1.176470588	3	106	107	37	4	3	3
51	45	42	-92	1.058823529	3	97	136	33.5	6	15	15
52	18	65	-73	1.176470588	5	70	158	35	6	19	14
53	86	54	-91	1.588235294	4	91	126	40	6	14	14
54	42	48	-122	1.764705882	3	106	113	41	6	15	15
55	33	47	-99	0.823529412	4	105	95	36	7	20	20
56	21	45	-120	1.235294118	4	92	129	45	9	18	17
57	23	49	-134	1.352941176	2	97	137	29.5	3	6	6
58	56	56	-76	1.588235294	5	113	94	40	7	8	8
59	97	100	-14	2	3	136	103	45	7	19	16
60	36	44	-148	1	3	103	118	38.5	6	10	10
61	41	37	-112	1.647058824	6	91	121	39.5	6	16	15
62	83	70	-86	1.764705882	6	112	62	42.5	7	15	15
63	61	51	-126	1.235294118	4	81	137	39	7	15	15
64	104	63	-99	1.323529412	1	115.5	96	34.5	4	5	5
65	34	44	-124	1.117647059	3	116	112	27	2	7	7
66	35	42	-173	1.352941176	3	94	124	31.5	4	3	3
67	52	49	-96	1.352941176	2	57	147	37.5	5	13	13
